# Dog agility tunnel risks for incidents

**DOI:** 10.3389/fvets.2025.1547824

**Published:** 2025-02-24

**Authors:** Dianne P. Ford, Kimberley L. Cullen, Kathryn Stickney, Murray Sharman, John D. Cullen

**Affiliations:** ^1^Faculty of Business Administration, Memorial University of Newfoundland, St. John’s, NL, Canada; ^2^School of Human Kinetics and Recreation, Memorial University of Newfoundland, St. John’s, NL, Canada; ^3^Form & Function – Veterinary Physioatherapy, Okehampton, United Kingdom; ^4^Department of Primary Industries, Brisbane, QLD, Australia; ^5^Faculty of Medicine, Memorial University of Newfoundland, St. John’s, NL, Canada

**Keywords:** dog agility, tunnels, risk factors, safety, competition

## Abstract

**Background:**

Flexible tunnels are the second most common obstacle on all dog agility courses, surpassed only by jumps. There has been a lot of debate and concern regarding risk factors associated with slips, falls and delayed exits (unseen slips, missteps, trips, falls). However, only one study was found which focused on the tunnel-related injuries, and it relied on handler reporting and did not consider base rates of the risk factors. As such, it is currently unknown which risk factors are statistically predictive of incidents. This study addresses this gap.

**Methods:**

Observational data from local, regional, national and international agility competitions (between June 30, 2023, to September 22, 2024) were collected from various agility organizations and countries by a team of researchers who are also judges and/or coaches within the sport. Tunnel, equipment, competition and course attributes, ground type and conditions along with tunnel incidents (slips, falls, and delayed exits) were recorded. Correlation, regression analyses, and chi-squared tests of independence were conducted to identify the relevant factors associated with incident rates.

**Results:**

The data included 563 tunnels (75.0% were incident free), with 30,418 tunnel performance observations (1.552% were incidents). The identified factors associated with incidents include tunnel characteristics (equipment specifications, shape on course), type and density of fixtures, course design (shape in design, angle of approach), ground and conditions. Their association with incident occurrence will be further detailed below.

**Discussion:**

Several previously assumed risk factors were relevant; however, some were not supported, and additional new factors were identified. Implications for future research and for organizations, judges, trial hosts, and competitors are discussed.

## Introduction

Since the 1970s when dog agility was first introduced in the UK, the sport has evolved significantly. Since then, the speed and performance criteria of obstacles have progressed, along with significant changes in the obstacles and environmental conditions. For example, the A-Frame has reduced in height, the surface of the contacts has changed, the nature and presence of slats on the contact equipment have evolved. Jump heights and design have also changed, and several obstacles have been eliminated from the vast majority of agility associations, including for example, the cross-over and chute (collapsible/collapsed/closed/flat tunnel).

A large motivation for the changes in the sport have been to improve safety for the canine athlete. Some of the changes have been evidence-based on research examining injuries and risk factors associated with the sport of agility, but many of the changes have been from anecdotal observations. A large amount of research has been handler/owner surveys on rates of injuries (1–8). More recently, research has examined the impacts of jumps and A-Frames on the dog (9–16). Similarly, the dogwalk and weave poles have been empirically examined (17–19).

Past research has identified several risks associated with injuries in agility through correlational research (self-reported data). The identified risks include: age of dog (3, 20), age of dog when started agility training (older had higher risk: 4; age when starting jumping, weaves, teeter training: 21), age of desexing (3, but not significant in all studies: 22), dog’s years of experience (2), previous injuries (2, 4), use of alternative therapeutic treatments (2), breed (border collies are higher risk: 2,3,5,22), level of competition (3, 21), contact with an obstacle (particularly A-Frame, dogwalk, jump: 1,5), jump height relative to shoulder height (15, 21), dry outdoor conditions (5), country (reported injuries in Australia > US: 21), handler experience (2), handler age (>65 lower risk: 22), handler occupation (dog trainer or not: 22), and handler medical training (22). Four survey studies on agility dog injuries (1, 4, 5, 8) have identified injuries associated with flexible (open) tunnels (of 1,209 ‘first reported injuries’, 17 mild and 12 severe injuries occurred due to direct contact with or falls in the tunnel: 1; 15.9% of the dogs were reported to have injuries associated with the open tunnel: 4; the open tunnel was the 7th most frequently cited obstacle associated with injuries: 5; and open tunnels accounted for 8.5% of reported digit injuries: 8).

The flexible tunnel is another obstacle that has garnered more attention recently for safety risks. The flexible tunnel can range from 10′/3 m to 20′/6 m and can be secured into a variety of shapes (from straight to curved formations). Apart from jumps, the flexible tunnel is the next most common obstacle in an agility course. It may comprise approximately 10–20% of the course’s obstacles that the dog must navigate, and it is commonly used to create a turn on a dog’s path to redirect them away from the edge of the ring. It may also be used to test certain skills or to accelerate the dog (23). Flexible tunnels have also evolved over time, with an increase in variety of lengths (shorter tunnels becoming more common), permissible shapes (S-shape is dominantly prohibited given the requirement for lead change inside the tunnel and tight turns), type and number of fixtures (increased number of fixtures and width of straps, weight of bags, and measures to ensure the tunnel remains stationary relative to the ground), type of interior (anti-slip grip), and density of wire pitch (becoming denser).

Given the frequency of interaction with this obstacle, it behooves researchers and agility experts to examine the factors that create risk for injury with the dog. Indeed, a recent survey found that various tunnel factors appeared to be associated with injury (23). Lott (23) examined handler-reported injuries of dogs and some of the known characteristics of the tunnel and conditions. The types of self-reported injuries associated with the tunnels included: shoulder injuries (28%) and iliopsoas strain (14%), slipped disc, torn ACL (“ALC [sic]”), and cuts were the least frequent (1.5% each) (23). This study did not investigate differences in injuries between breeds, but Lott (23) noted it may play a factor given speed, shape, size and weight. However, past research has found border collies to have a higher risk of injury in agility relative to non-border collies (2, 3, 5, 15), but these studies did not examine the interplay between breed and obstacles for injuries aside from border collies and jump heights (15).

Lott (23) reported that the extent the tunnel had anti-slip interior (none, half, full), the ring surface (grass, sand, artificial turf), ground conditions (wet, dry), darkness of the tunnel colour, the fixtures, damaged or poor quality of tunnels appear to have a role in injury risk. Of note, Lott’s survey found that the bracket or parenthesis shape, which is commonly referred to as “(−shaped” tunnel had the highest proportion of slips/falls (44%) and the fewest slips/falls occurred on the straight (2%) or gently curved (8%) tunnels (23).

A limitation of Lott’s (23) study is that it was based on handler, judge and trainer surveys and did not did not account for how often each tunnel shape was used or how frequently dogs navigated them without incident, limiting the ability to assess relative risk accurately. Thus, it was not clear if the proportion of incidents was unexpectedly high for a particular tunnel shape or if that tunnel shape was also proportionally the most common shape used. It also relies on self-reported data, which is susceptible to recall error and confirmation bias (i.e., misattributing the cause to an expected factor while missing an unexpected factor). Accordingly, it is important to not make conclusions about overall risk or overstate implications, and to investigate this obstacle’s risk factors further.

To accomplish this, we conducted an international study in which the dog injury-risk performances (i.e., slips, falls, delayed exits) along with good performances (i.e., no slip, fall or delayed exit) were recorded along with all identifiable traits of the tunnels. The purpose of our study was to determine predictors of injury-risk incidents. Injuries were not directly measured as there may be a delayed effect with sprains and contusions, and dogs might not show lameness in the higher arousal state of running the course. In addition, dog performance failures (i.e., refusals) were not coded as these have not been empirically linked to risks of subsequent injury. Refusals could indicate an existing injury; however, there are many possible reasons for refusals, including training and foreign objects/scents inside the tunnel.

Tunnels vary in terms of length, the material (amount of grip inside the tunnel), the wire pitch (the distance between the wires that give it structure), the colour, the colour pattern, and its age/condition. Outside the tunnel, there are also a variety of fixtures to secure the tunnel (e.g., cinch-type screw-in fixtures, sandbags of a variety of shapes and fillings, and plates), density of fixtures (i.e., length of tunnel per fixture), and the tunnel’s set shape. Additionally, it has been surmised that the ring surface (e.g., grass, sand, turf) may play a role in how a tunnel functions and also the conditions of the ground (dry, wet, muddy, standing water). Functionally, there is also the location of the tunnel within the course (i.e., obstacle number); also, it may vary in terms of location on the dog’s path (i.e., straight entry, angled-open, blind entry). Finally, it may vary in terms of what is the dog’s expected lead leg as they enter the tunnel (same as the curve, opposite to the curve).

In agility, equipment and course specifications are overseen by different sanctioning organizations (e.g., Fédération Cynologique Internationale, UK Agility International, national agility associations, and national kennel clubs). The exact specifications may vary across these organizations, but the focus for this study was to capture the characteristics of the tunnels and their situation in a course.

All of the above factors may play a role or have interaction effects that impacts the dog’s risks of slipping, falling or having a delayed exit (i.e., possibly slipping/falling inside the tunnel unseen by the handler or judge). The most common factors seen in social media or agility forums are the following:

1) The level of traction inside the tunnel – tunnel interior (e.g., anti-slip grip), conditions (e.g., wetness) and ground (e.g., loose sand) appear to be the most commonly mentioned risks for slips and falls.
2) The length and shape of the tunnel – while the S-shape is officially prohibited in most (if not all) organizations, there are also J, L, U-shaped tunnels that are possible and often discussed. C-shaped tunnels are starting to lose favor as well, with straight, gentle curves, and (−shaped most heavily promoted for safety (see Supplemental Materials Figure A for illustration guide of these shapes). Similarly, there are contingents who dislike the 10′/3 m tunnel with any bend added to that shape, ostensibly due to the higher speeds dogs achieve in the shorter tunnel.
3) The colour of the tunnel – the general discussion is that dark tunnels (e.g., dark blue) are worse than light-coloured tunnels (e.g., yellow), arguably due to lower visibility for the dog.
4) The type and density of fixtures on the tunnel – while there is no consensus on screw-in cinches versus sandbags, there appears to be a consensus that more secure is better (e.g., higher fixture density, and fixtures that will not give way to the dog with force like plates or screw-in cinches). There is also some concern about the increased number of fixtures (i.e., higher fixture density) negatively impacting visibility.
5) The angle of approach to the tunnel – some judges are promoting a straighter approach than angled approach to the tunnels to reduce risks of incidents.

All of these factors were explored empirically, along with all of the other factors that have not yet been examined, such as wire pitch, dog’s lead leg on approach, location in the course (obstacle number), event level and class level. We also examined for interaction effects to test the above assertions. To that end, we do not present any formal hypotheses, aside from the following research question: *Which tunnel, design, environmental, experiential factors (if any at all) have a predictive relationship to injury-risk incidents?* Altogether, this research provides more information regarding risk factors associated with the tunnel and how it is used in agility. The results may inform equipment and course standards for organizations, course design for judges and trainers, course analysis for competitors and equipment purchases for manufacturers, trainers, hosts, and competitors.

## Materials and methods

### Procedure

The methodology for this study was tunnel-level analysis, such that each tunnel was a subject (a course with three tunnels in it would provide the study with three subjects for data collection), with observational data (tunnel, course, ring and design details and dog performance) and document data (course maps if available) collected by coders.

#### Coders and coding criteria

There were five coders with four additional assistant coders. The five coders were all trained researchers in a variety of fields and have extensive agility training background (e.g., coaches, judges, world-team competitors). The four assistant coders were experienced in the sport of agility (agility coaches, high level competitors) and were blind to the specific research questions. They aided three of the coders when they were unavailable for some of the runs. The coders did not seek to alter any of the conditions of the tunnels on the course; data was solely observational in public accessible events.

The five researchers met virtually and discussed all of the coding criteria and deliberated on the standards for the codes. A coding sheet to aid in classifying characteristics of the tunnel, design elements, etc. was created (see Supplemental Materials). A second virtual discussion was held when the shape of the tunnel needed better distinction between some of the loose curves (e.g., the (−shape tunnels. To address that, the lead author developed a visual graph with assessment criteria that could be distinguished visually in person and through video. Specifically, three shape codes were created: (1) gentle curved tunnels have a straight line that runs from one exit to the other; (2) (−acute are (−shaped tunnels where the refusal planes create an acute angle; and (3) (−obtuse are (−shaped tunnels where the refusal planes create an obtuse angle when they intersect (see Supplemental Materials Figure A).

#### Coding procedure

One to two coders coded each run. When two were present, any questions on coding were discussed. In all instances, consensus was immediately achieved, which indicates there was consistency among coders on how to code the data. Coding involved two data entry tables (one for tunnel case characteristics and one for dog performances for each tunnel), and was coded either directly into online spreadsheets or collected on pen and paper and later transcribed electronically. Course maps, when available, were recorded along with the data and stored with the respective coder(s).

Before each course was run, the researcher(s) coded the characteristics of each tunnel on the course. Then during the running of the course, each dog’s performance was recorded for each tunnel. The dog’s performance was coded as one of the four potential outcomes, all of which were mutually exclusive and comprehensive: (1) Good, (2) Slips, (3) Falls, (4) Delayed Exits. The variable Slips included all visible slips, trips and missteps. Falls included all visible shoulder, hip or full-body contact either onto the wall/floor of the tunnel or onto the ground just outside the tunnel. Delayed Exits included all unseen incidents and discernable delayed exits. Good was coded if none of the other outcomes were present (i.e., no incident occurred).

Once the course was completed, each tunnel’s outcome codes were counted and recorded (e.g., 54 good performance, 1 slip, 0 falls, 3 delayed exits; with 58 attempts on that one tunnel). Thus, for any given course, each tunnel might not have the same total count due to “missing” tunnel observations.

#### Exclusions from coding

Refusals were not coded as these were not of interest. However, if the coder noted there was a collision inside the tunnel (as seen through tunnel wall movements or heard from the outside), then it was coded as a delayed exit regardless of which tunnel mouth the dog exited. In addition, tunnels that were taken out of sequence were not recorded given the approach and lead were not as designed in the course and would introduce measurement error to the analysis. If the dog was pulled from the run or missed the tunnel entirely, or if the coder could not fully see the performance of the tunnel, then that observation was recorded as missing. Missing observations were not included in the analysis.

### Sample

Events were chosen due to availability to the coders, and accessible for public observation. Given the events were public observations, ethics clearance was not required for this research as per TCPS2 Guidelines. This included local, regional, and national events being coded in person in three different countries with multiple agility organizations: Canada (multiple provinces; AAC, UKI, CKC), United Kingdom (KC), Australia (ANKC, ADO). In addition, world-level events and tryout-level (or equivalent) were coded through video coverage and included Junior Agility World Championship (England), European Open (Denmark), World Agility Open (Netherlands), Agility World Championship (Czechia), Team Canada Tryouts (Canada), Canada Open (Canada), and West Coast Open (United States).

### Analyses

First interrater reliability was tested, then the data was analysed for descriptive statistics. Next the outcome variables were converted from counts to percentages (% good, % slip, % fall, % delay) such that for each tunnel, the percentages would add to 100%. This conversion was necessary as these outcome variables are mutually exclusive (if the dog has a good performance, they could not have one of the other three outcomes). Furthermore, we needed to adjust for the fact that some classes were substantial in size (over 200 dogs) and some were very small (5 dogs or fewer). By converting to percentages, each tunnel carried equal weight in the analysis. The outcome variables were then tested for skewness and kurtosis. If there was non-normal data (skewed and/or with kurtosis), one more conversion would be completed to normalize the outcome variables to enable regression analysis.

Prior to any correlation or regression analyses, some predictor variables needed to be recoded. Specifically, dummy variables for nominal data that did not have meaningful ordinal meaning were created, and a fixture density variable was calculated. Fixture Density was created by computing the number feet (of tunnel) per fixture. This was then translated into a categorical variable based on four meaningful categories based on the algorithm of “1 fixture per meter length plus 1 more fixture.” With 3, 4, 5 and 6 m options, the densities translate to 2.5, 2.6, 2.67 and 2.86 feet per fixture, respectively. Thus, the following were created: “low density” (2.87–4′/fixture), “low common density” (2.52–2.86′/fixture), “common density” (2.50–2.51′/fixture), “high density” (2.01–2.49′/fixture), and “very high density” (1.50–2.00′/fixture). Once these conversions were completed, we conducted a Pearson correlation table of all predictor and outcome variables, then multiple regression analyses to assess potential interaction effects to address the five common factors as discussed above. Graphical representations of significant interaction effects were also created.

## Results

### Interrater reliability

Interrater reliability was determined several ways. First, the two main coders coded a full class together, which included 282 cases, 564 decisions, and had an initial percent agreement of 95.7% (Cohen’s kappa = 0.885), which is considered “almost perfect agreement” (24). Points of disagreement were discussed and clarity on codes were achieved. The international coders were then tested with the lead coder for interrater reliability. To do this a selection of run videos were compiled that were partially random (192 tunnel observations were randomly selected from an event that was not included in the data set, and 166 tunnel observations were a combination of selected due to known incident and others that did not necessarily have a known incident). Coders watched the videos and coded independently. This resulted in 1264 decisions (some tunnels were not clear for coding and were deemed missing). The observed pairwise agreement was 94.6% which was above the expected agreement of 87.7% (Fleiss’ kappa = 0.562; moderate agreement). The lead author reviewed the disagreements and there was no single individual who disagreed more than others, and there was no particular incident code that was problematic for agreement. Finally, the lead researcher’s test–retest reliability was conducted on a single course that was videoed and achieved 97.6% agreement (Cohen’s kappa = 0.929; almost perfect agreement).

### Descriptives and Pearson correlations

#### Sample descriptives

Every level of competition was included from the lowest levels (Beginner/Grades 1-3/Starters/Novice) to the highest levels (Champion/Grade 7/Masters/World’s) (see Appendix A for breakdown of the descriptives of the sample). There was a total of 19 events, 151 courses, 563 tunnels, for an average of 3.73 tunnel completions per course, and a total of 30,418 recorded tunnel performances.

#### Variables: descriptive analyses

The descriptive statistics can be seen in Table 1. In the 30,418 tunnel performances across the 563 tunnels, there were 472 incidents (243 Slips, 53 Falls, 176 Delayed Exits) and 29,946 Good. This gives us the frequency rate of 1.552% of all on-course tunnel performances involved an incident (of the performances, 0.8% were slips, 0.2% were falls, and 0.6% were delayed exits). However, the frequency of incidents was not random across all tunnels. There were 420 (74.6%) tunnels which had 100% Good, 98 (17.4%) tunnels with Slips, 36 tunnels (6.4%) tunnels with Falls, and 72 tunnels (12.8%) with Delayed Exits.

**Table 1:** Variable descriptive statistics and Pearson correlations to the outcome variables: Z%Good = Z-score of percent good performances, Z%Slip = Z-score of the percent slip outcomes, Z%Fall = Z-score of percent fall outcomes, Z%Delay = Z-score of the percent delayed exit outcomes.

	Mean	SD	*N*	Z%Good	Z%Slip	Z%Fall	Z%Delay
Zscore (PercentGood)	0.00	1.00	563	---			
Zscore (PercentSlip)	0.00	1.00	563	**−0.605** ^**^	---		
Zscore (PercentFall)	0.00	1.00	563	**−0.422** ^**^	**0.378** ^**^	---	
Zscore (PercentDelay)	0.00	1.00	563	**−0.812** ^**^	0.061	0.062	---
Event level	2.13	1.25	564	−0.075	0.068	−0.018	0.059
Class level	3.02	0.95	564	−0.074	0.042	−0.063	**0.085** ^*^
Obstacle number	10.43	5.22	564	0.002	−0.014	−0.047	0.019
tunnel age	0.82	1.61	405	0.085	−0.055	−0.041	−0.066
# of colours	1.04	0.25	560	−0.011	−0.018	**0.130** ^**^	−0.006
Tunnel colour							
Yellow	0.40	0.60	560	0.036	−0.032	−0.027	−0.020
Light blue	0.06	0.24	560	0.059	−0.064	−0.045	−0.025
Light purple	0.12	0.32	560	0.010	−0.080	−0.037	0.047
Light pink	0.10	0.30	560	0.012	−0.052	0.031	0.009
Green	2.80	3.26	560	−0.034	**0.097** ^*^	0.023	−0.023
Red	1.40	2.60	560	**−0.087** ^*^	**0.132** ^**^	0.081	0.010
Dark blue	0.54	1.87	560	0.001	0.007	0.069	−0.023
Dark purple	0.04	0.19	560	0.025	−0.001	−0.036	−0.024
Black	0.02	0.13	560	0.005	−0.036	**0.145** ^**^	−0.019
Tunnel interior							
No anti-slip	0.15	0.36	564	0.006	0.044	0.023	−0.041
Half anti-slip	0.02	0.13	564	0.008	−0.038	**0.135** ^**^	−0.020
Full anti-slip	0.83	0.38	564	−0.008	−0.029	−0.069	0.046
Tunnel shape							
Straight	0.27	0.45	564	0.055	**−0.111** ^**^	−0.056	0.012
Gentle (exits visible)	0.22	0.42	564	0.021	0.016	0.053	−0.049
(−acute	0.29	0.45	564	0.042	−0.027	−0.044	−0.027
(−obtuse	0.15	0.35	564	−0.051	0.053	0.004	0.032
C-shape	0.01	0.12	564	0.026	−0.034	−0.022	−0.007
U-shape	0.01	0.07	564	−0.025	0.074	−0.013	−0.011
J-shape	0.03	0.18	564	**−0.137** ^**^	**0.087** ^*^	**0.131** ^**^	**0.091** ^*^
L-shape	0.01	0.10	564	−0.058	**0.089** ^*^	−0.019	0.023
S-shape	0.01	0.07	564	−0.073	**0.145** ^**^	0.057	−0.011
Location of vertex	0.13	0.54	564	**−0.267** ^**^	**0.259** ^**^	**0.155** ^**^	**0.145** ^**^
Length of tunnel	16.85	3.11	564	**−0.142** ^**^	**0.102** ^*^	0.041	**0.111** ^**^
Wire pitch spacing	5.17	1.07	547	0.059	−0.034	0.050	−0.068
Length per fixture	2.36	0.41	564	0.008	0.077	−0.020	−0.054
# of fixtures on tunnel	7.33	1.74	564	**−0.120** ^**^	0.023	0.046	**0.130** ^**^
# of extra bags behind	0.00	0.08	564	−0.021	0.034	−0.008	0.008
Type of fixture							
Mouth cinch	0.12	0.32	564	0.022	0.016	0.014	−0.042
Mouth sandbag wide	0.70	0.46	564	**0.151** ^**^	**−0.108** ^*^	−0.044	**−0.118** ^**^
Mouth sandbag narrow	0.01	0.10	564	0.030	−0.029	−0.019	−0.016
Mouth plate	0.01	0.10	564	0.030	−0.029	−0.019	−0.016
Body cinch	0.10	0.29	564	−0.011	0.038	**0.099** ^*^	−0.035
Body sandbag wide	0.90	0.29	564	0.011	−0.038	**−0.099** ^*^	0.035
Body sandbag narrow	0.11	0.31	564	−0.010	0.038	0.030	−0.019
Water Bag	0.01	0.07	564	−0.034	−0.021	**0.276** ^**^	−0.011
Shape of tunnel bag							
Saddlebag	0.14	0.35	516	−0.013	0.041	0.062	−0.023
Cylinder	0.01	0.08	516	0.021	−0.020	−0.013	−0.012
Triangle	0.99	0.11	516	−0.010	0.029	0.018	−0.009
Ground (footing)							
Grass	0.54	0.50	564	**0.148** ^**^	**−0.087** ^*^	−0.002	**−0.137** ^**^
Sand	0.28	0.45	564	−0.039	−0.005	−0.008	0.056
Artificial turf	0.18	0.38	564	**−0.147** ^**^	**0.119** ^**^	0.012	**0.112** ^**^
Conditions							
Dry (not burned)	0.65	0.48	564	−0.025	−0.027	−0.067	0.066
Damp	0.22	0.41	564	0.019	0.010	**0.085** ^*^	−0.052
Wet (actively raining)	0.10	0.30	564	0.024	0.004	−0.004	−0.033
Soaked	0.02	0.14	564	−0.033	0.064	−0.004	0.003
Muddy	0.01	0.08	564	0.017	−0.024	−0.015	−0.004
Approach to tunnel							
Straight approach	0.40	0.49	564	0.071	−0.067	−0.050	−0.038
Straight-open approach	0.03	0.18	564	0.028	−0.013	−0.017	−0.024
Open angled approach	0.49	0.50	564	−0.033	0.066	0.050	−0.011
Refusal plane approach	0.03	0.16	564	**−0.195** ^**^	0.062	0.030	**0.207** ^**^
Blind entry	0.06	0.24	564	0.036	−0.035	−0.011	−0.022
Expected lead on approach							
Same lead	0.57	0.49	564	−0.080	**0.129** ^**^	0.064	0.006
Opposite lead	0.21	0.41	564	−0.009	−0.033	0.009	0.030
Expected exit angle	1.86	1.00	176	0.065	−0.027	−0.015	−0.062

Bold numbers are statistically significant: *p < 0.05, ** *p* < 0.01.

As expected, the outcome variables were highly skewed with high kurtosis. The distribution for Good was left-skewed and had a very high peak (skew/kurtosis: -0.810/104.913). The distributions for Slips (7.772/91.031), Falls (7.713/67.025), and Delayed Exits (15.323/272.674) were right-skewed with very high peaks. These distributions indicate that these incidents have a very low base rate and are not normally distributed.

#### Variable Pearson correlations

Table 1 also presents the correlation matrix of the research variables. Of note, Slips and Falls were correlated (*r* = 0.378, *p* < 0.01), and delayed exits were not correlated with either Slips (*r* = 0.061, *p* > 0.05) or Falls (*r* = 0.062, *p* > 0.05).

### Simple linear regression analyses

Given the outcome variables were skewed with kurtosis, they were standardized for regression. The predictor variables were not standardized as they were categorical/dummy variables or did not violate normality of error terms.

The results of the regression analyses indicated that there were significant predictors of Good outcomes (*F*_(42,296)_ = 2.201, *p* < 0.001, *R^2^_adj_* = 0.130). The statistically significant predictors comprised of the following: tunnel shape (U-curve: *β_adj_* = 0.182, *p =* 0.017), vertices in tunnel shape (middle: *β_adj_* = −0.182, *p* < 0.001; end: *β_adj_* = −0.300, *p =* 0.027), and approach to entry (refusal plane approach: *β_adj_* = −0.179, *p* = 0.003) (see Table 2 for summary of the regression model).

**Table 2:** Simple linear regression of potential predictors of good performances, slips, falls and delayed exits.

	Good (β_adj_)	Slips (β_adj_)	Falls (β_adj_)	Delayed (β_adj_)
***F*-value**	2.201***	1.880***	1.193	1.807**
**(df1, df2)**	42, 296	42, 296	42, 296	42, 296
**R** ^**2**^_**adj**_	0.130	0.099	0.023	0.091
Variables				
Event level	−0.008	0.169	−0.005	−0.082
Class level	0.049	−0.075	−0.014	−0.016
Tunnel obstacle # on course	0.003	−0.053	−0.073	0.039
Tunnel age	0.209	−0.227	−0.177	−0.099
# of colours on the tunnel	−0.004	−0.058	0.027	0.032
Tunnel colour				
Yellow	0.081	−0.177	0.004	−0.002
Light blue	−0.034	0.088	−0.024	−0.002
Light purple	0.051	−0.057	−0.036	−0.025
Light pink	0.009	−0.012	0.016	−0.008
Green with white stripe	0.040	0.001	−0.038	−0.042
Red	−0.023	0.354*	0.063	−0.178
Dark blue	Removed	Removed	Removed	Removed
Dark purple	0.066	−0.381*	−0.133	0.154
Yellow top, black bottom	Removed	Removed	Removed	Removed
Tunnel Interior				
No anti-slip	Removed	Removed	Removed	Removed
Half anti-slip	Removed	Removed	Removed	Removed
Full anti-slip	0.238	−0.293	−0.186	−0.097
Tunnel Shape				
Straight	Removed	Removed	Removed	Removed
Gentle curve (visible exit)	0.106	0.020	−0.140	−0.115
(−acute	0.033	0.023	−0.119	−0.031
(−obtuse	−0.063	0.230*	0.045	−0.059
C curve	0.020	−0.019	−0.099	0.004
U curve	0.182*	0.075	−0.079	−0.250***
J curve	Removed	Removed	Removed	Removed
L curve	0.105	0.152*	−0.022	−0.209**
S curve	Removed	Removed	Removed	Removed
Vertices				
No vertex	Not Entered	Not Entered	Not Entered	Not Entered
Vertex at entry	−0.037	−0.007	0.050	0.039
Vertex in middle	−0.380***	0.051	0.021	0.436***
Vertex at exit	−0.130*	0.167**	0.184**	0.033
Tunnel length	−0.087	0.010	−0.003	0.102
Wire pitch	0.037	0.142	0.300	−0.181
Number of fixture sets	Not Entered	Not Entered	Not Entered	Not Entered
Feet of tunnel per fixture	−0.026	−0.002	−0.049	0.043
Split-bags	Removed	Removed	Removed	Removed
Extra bags	Removed	Removed	Removed	Removed

Entry/exit fixture types:				
Ends: cinch-screwed into ground	0.081	−0.188	−0.230	0.047
Ends: tunnel bags with peagravel/sand (wide strap)	0.130	−0.416	−0.437	0.152
Ends: tunnel bags with peagravel/sand (narrow strap)	Removed	Removed	Removed	Removed
Body: cinch—screwed into ground	Removed	Removed	Removed	Removed
Body: tunnel bags with peagravel/sand (wide strap)	0.008	0.016	0.006	−0.019
Body: tunnel bags with peagravel/sand (narrow strap)	Removed	Removed	Removed	Removed
Body: tunnel bags with water	Removed	Removed	Removed	Removed
Fixture style: saddle-bag style	−0.012	0.017	0.026	0.000
Fixture style: rounded/cylinder	0.030	0.023	0.022	−0.054
Fixture style: triangle style	−0.032	−0.013	−0.010	0.048
Ground (footing):				
Grass surface	Removed	Removed	Removed	Removed
Sand surface	−0.155	−0.085	−0.074	0.251
Artificial turf	−0.069	−0.195	−0.023	0.197
Conditions:				
Dry	−0.067	−0.040	−0.178	0.138
Damp to wet, no active rain	Removed	Removed	Removed	Removed
Actively raining, wet ground	0.015	−0.040	0.007	−0.010
Soaked with standing water	−0.007	−0.040	−0.035	−0.002
Muddy tracks	Removed	Removed	Removed	Removed
Approach:				
Straight approach	Removed	Removed	Removed	Removed
Handler choice/dog path variance in design results in either straight or angled-open	0.031	−0.003	0.007	−0.038
angled-open (not past refusal plane)	−0.061	0.098	0.052	0.012
Handler choice/dog path variance in design results in either angled-open or closed (blind) approach	−0.179**	−0.014	−0.108	0.248***
closed (blind) approach	−0.030	−0.002	0.003	0.037
Expected lead on approach				
n/a—straight tunnel	Removed	Removed	Removed	Removed
Same lead as tunnel	−0.081	0.071	0.169	0.029
Opposite lead as tunnel	−0.045	−0.012	0.107	0.042
Unknown lead (handler choice)	Removed	Removed	Removed	Removed

For all models, the variables noted as “removed” in the table are constants or have missing correlations and were deleted from the model. Bold numbers are statistically significant: * *p* < 0.05, ** *p* < 0.01, *** *p* < 0.001.

The regression for Slips explained 9.9% of the variance (*R^2^_adj_*), and was statistically significant (*F*_(42,296)_ = 1.880, *p =* 0.001). The statistically significant predictors comprised of the following: tunnel colours (red: *β_adj_* = 0.354, *p* = 0.032; dark purple: *β_adj_* = −0.381, *p* = 0.012), shape of the tunnel (L-shape: *β_adj_* = 0.152, *p* = 0.041; (−obtuse: *β_adj_* = 0.230, *p* = 0.042), vertices in tunnel shape (end: *β_adj_* = 0.167, *p* = 0.005).

Delayed Exits (*F*_(42,296)_ = 1.807, *p <* 0.003, *R^2^_adj_* = 0.091) were predicted by the following: tunnel shape (U-curve: *β_adj_* = −0.250, *p* = 0.001; L-curve: *β_adj_* = −0.209, *p* = 0.005), presence of vertices (middle: *β_adj_* = 0.436, *p* < 0.001), and approach to tunnel (refusal plane trajectory: *β_adj_* = 0.248, *p* < 0.001). The regression for Falls was not statistically significant (*F*_(42,296)_ = 1.193, *p =* 0.204, *R^2^_adj_* = 0.023).

### Interaction tests for common safety assertions

#### Level of traction inside tunnels: tunnel interior, ground and conditions

**Figure 1:**
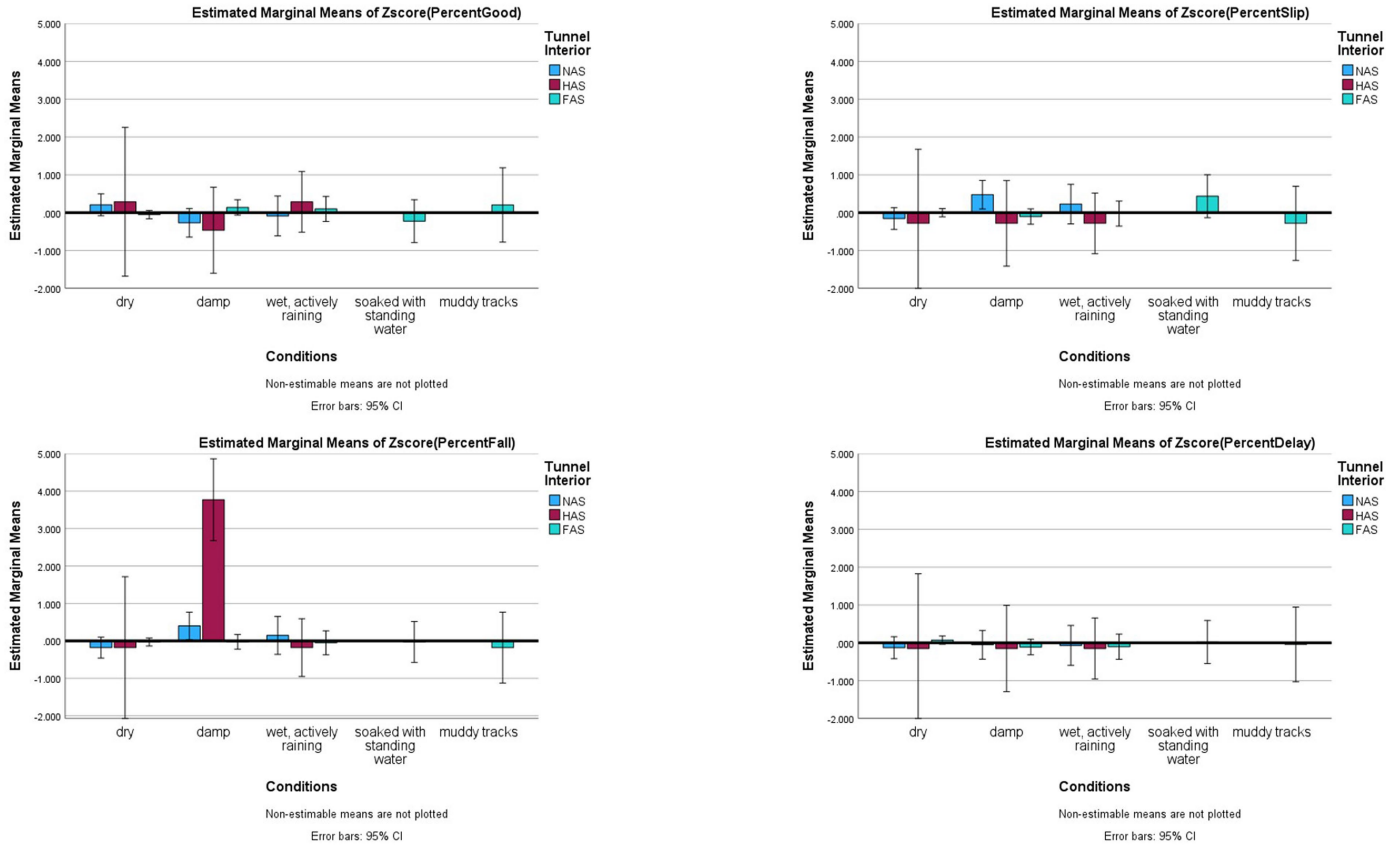
Risk factor averages for ground conditions by tunnel interior with 95% confidence interval error bars included. Tunnel interiors are: NAS = No Anti-Slip grip, HAS = Half Anti-Slip grip, FAS = Full Anti-Slip grip.

**Figure 2:**
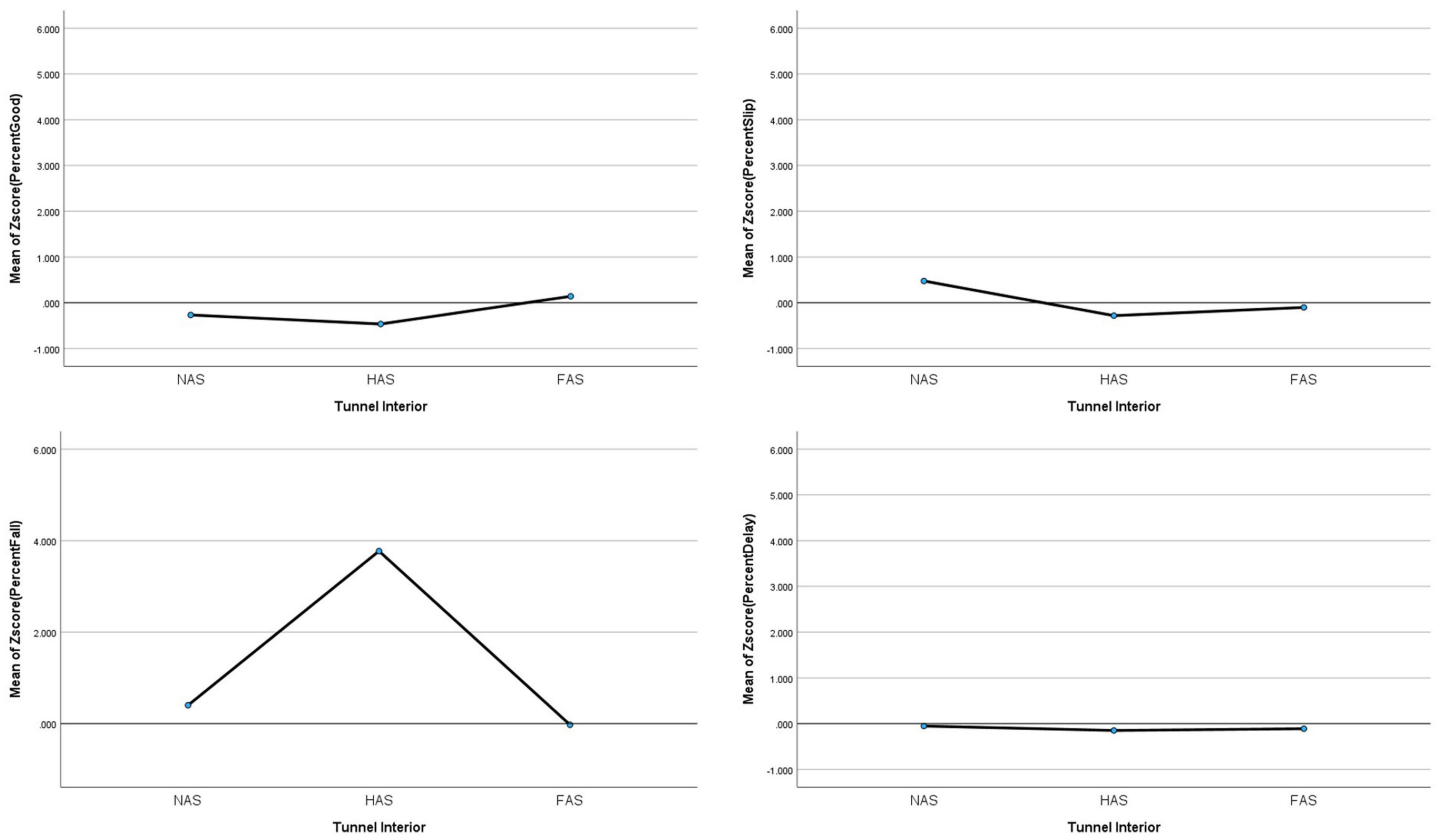
Averages for the different tunnel interiors in damp conditions as a risk factor in dog agility competition. Tunnel interiors are: NAS = No Anti-Slip grip, HAS = Half Anti-Slip grip, FAS = Full Anti-Slip grip.

To test potential interaction effects as suggested in the popular assertions, specific regressions were computed. For Assertion #1 regarding tunnel interior interactions with surface or conditions, two interaction effects were tested. Unfortunately, there were no “no anti-slip” or “half anti-slip” interiors available on sand or turf in the data set; thus, the interaction effects could not be tested fully. However, there were sufficient samples to test Tunnel Interior x Conditions, which had significant interaction effects only for Falls (*F*_(4,552)_ = 9.378, *p* < 0.001, *partial eta^2^* = 0.064) where half anti-slip tunnels had significantly more Falls than no anti-slip or full anti-slip in the damp condition. However, Good, Slips, and Delayed Exits did not have significant interactions between tunnel interior considering all conditions (*p >* 0.05; see Figure 1). When only the Damp condition was examined (second most common and often a concern for competitors), significant differences were found between no anti-slip and full anti-slip tunnels for Good (*F*_(2,120)_ = 6.403, *p* < 0.001, *eta^2^ =* 0.096, NAS: −0.267 ± 0.942, FAS: 0.140 ± 0.140), and Slips (*F*_(2,120)_ = 5.896, *p* = 0.004, *eta^2^ =* 0.089, NAS: 0.475 ± 1.473, FAS: −0.103 ± 0.437). Falls had significant differences: half anti-slip tunnels had more incidents than full anti-slip and no anti-slip tunnels, but no significant differences were found between no anti-slip and full anti-slip tunnels (*F*_(2,120)_ = 9.634, *p* < 0.001, *eta^2^ =* 0.138, HAS: 3.771 ± 6.842, NAS: 0.402 ± 1.788, FAS: −0.025 ± 1.024; see Figure 2).

**Figure 3:**
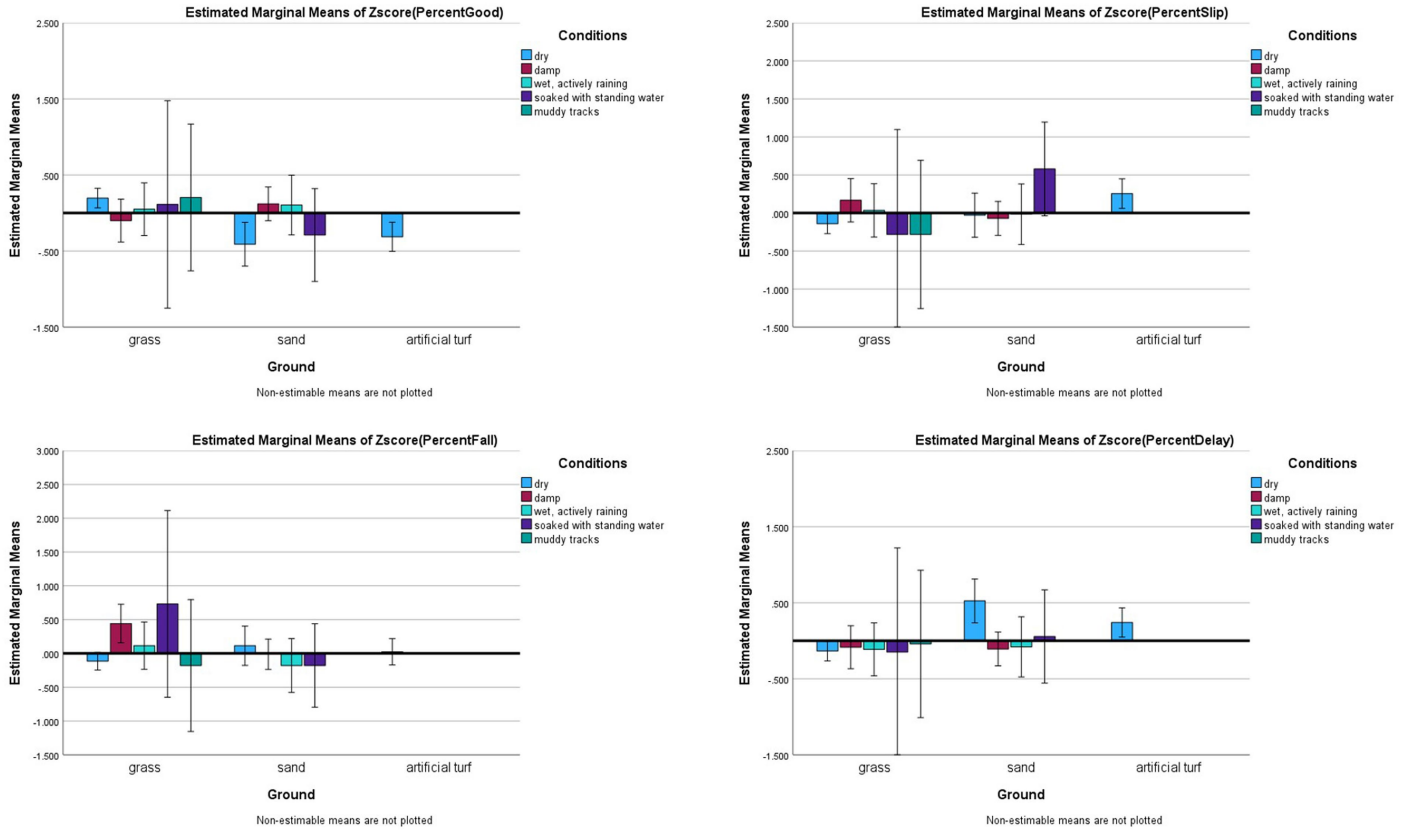
Risk factor averages for type of ground by conditions with 95% confidence interval error bars included.

**Figure 4:**
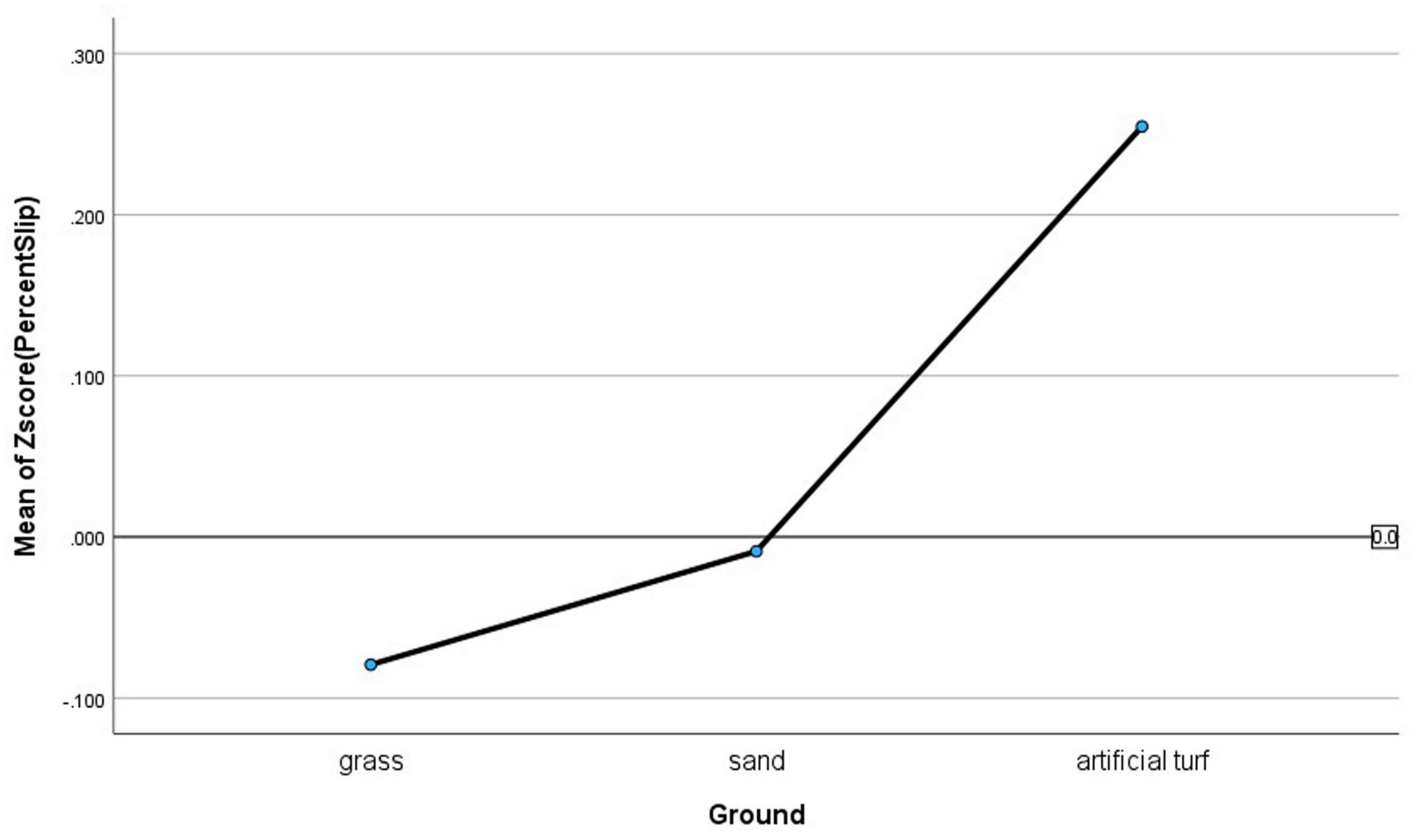
Averages for the different ground types as a slip in tunnels risk factor in dog agility competition.

Another related interaction was Ground x Conditions given common beliefs suggest that this may be a relevant interaction for incidents with tunnels. The results support this assertion, such that the main effects for Ground and Condition were not significant for Good, Falls, and Delayed Exits, but the interaction effects were (Good: *F*_(3,553)_ = 4.243, *p =* 0.006, *partial eta^2^* = 0.023; Falls: *F*_(3,553)_ = 3.128, *p =* 0.025, *partial eta^2^* = 0.017; Delayed Exits: *F*_(3,553)_ = 3.042, *p* = 0.029, *partial eta^2^* = 0.016). Slips were not significantly predicted by Ground x Conditions *(F*_(3,553)_ = 1.128, *p* = 0.337, *partial eta^2^ =* 0.006) but this outcome was predicted by the main effect for Ground (*F*_(2,553)_ = 4.009, *p* = 0.018, *partial eta^2^ =* 0.014), such that artificial turf (0.255 ± 0.17) had more Slips than grass (0.009 ± 0.76). No other group differences were found for Slips (see Figure 3 for interaction effects illustrations and Figure 4 for main effect for Ground with Slips).

For good performance, there were significant differences between dry (not burned) grass outperforming dry sand (*p* = 0.003) and dry artificial turf (*p* < 0.001). Dry grass also outperformed damp grass (*p* = 0.002). For falls, dry grass had fewer falls than damp-wet grass (*p* = 0.004), but there were no other significant group differences on these interactions (*p* > 0.01). Dry grass had fewer delayed exits than dry sand (*p* = 0.003). There were no other significant group differences with Bonferroni correction considered.

#### Length and shape of tunnels

**Figure 5:**
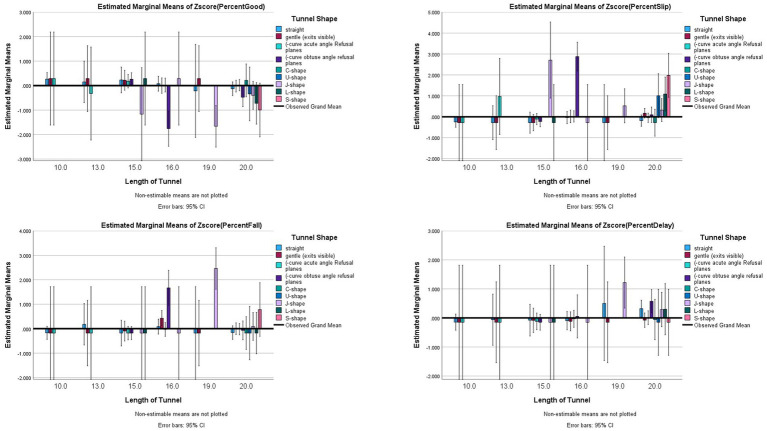
Risk factor averages for tunnel length by tunnel Shape with 95% confidence interval error bars included.

To test Assertion #2, regarding the length and shapes of the tunnels, Length x Shape interaction was tested. The results showed significant interaction effects (Good: *F*_(15,534)_ = 1.831, *p* = 0.028, *partial eta^2^* = 0.049; Slips: *F*_(15,534)_ = 4.661, *p <* 0.001, *partial eta^2^* = 0.116; Falls: *F*_(15,534)_ = 2.027, *p* = 0.012, *partial eta^2^* = 0.054); however, for Delayed Exits, no significant interaction was found between length and shape (*F*_(15,534)_ = 0.563, *p* = 0.903, *partial eta^2^* = 0.016) (see Figure 5 for illustration).

For 10′ and 13′ tunnels (3 m, 4 m), there were no significant differences between shapes (regardless of straight or gentle curve with the exit visible) for all outcomes (*p* ≥ 0.30). It should be noted that for the 10′ (3 m) tunnels, there were 3,042 performances, and only 8 incidents (6 Slips, 1 Fall, 1 Delayed Exit), all of which were on dry, straight, 4″ pitch, full anti-slip tunnels that were secured with cinches as there were no other differences on tunnel/course/grounds characteristics for the perfect performing 10′ (3 m) tunnels. For 13′ (4 m) tunnels, there were 510 performances and 3 incidents, all in very wet conditions (no other unique characteristics).

There were no significant differences for the 15′ tunnels (4.6 m) across shapes for good performances or falls, but there were more Slips for the J-shape than the straight, gentle curve with visible exits (being able to see through the tunnel) or (−shapes (*p* < 0.013). L-shaped 15′ (4.6 m) tunnels did not differ from all shapes (*p >* 0.013).

For 16′ (5 m) tunnels, the (−obtuse had significantly less Good outcomes (*p <* 0.013) than (−acute, and gentle curves with exit visible. The (−obtuse also had more Slips than all other observed shapes (*p* < 0.013), and had more Falls than straight, gentle curve with visible exits, or (−acute tunnels (*p <* 0.013). The 19′ tunnels (5.8 m) had no significant group differences for Good or Slips (*p >* 0.013), but did have more Falls than gentle curve with visible exit tunnels (*p* < 0.013).

For 20′ (6 m) tunnels, S-shaped tunnels had significantly more Slips (*p* < 0.013) than straight, (−acute, C-shaped tunnels. There were no significant group differences between shapes for Good or Falls for the 20′ (6 m) tunnels (*p* > 0.013).

#### Colour of the tunnel: visibility

**Figure 6:**
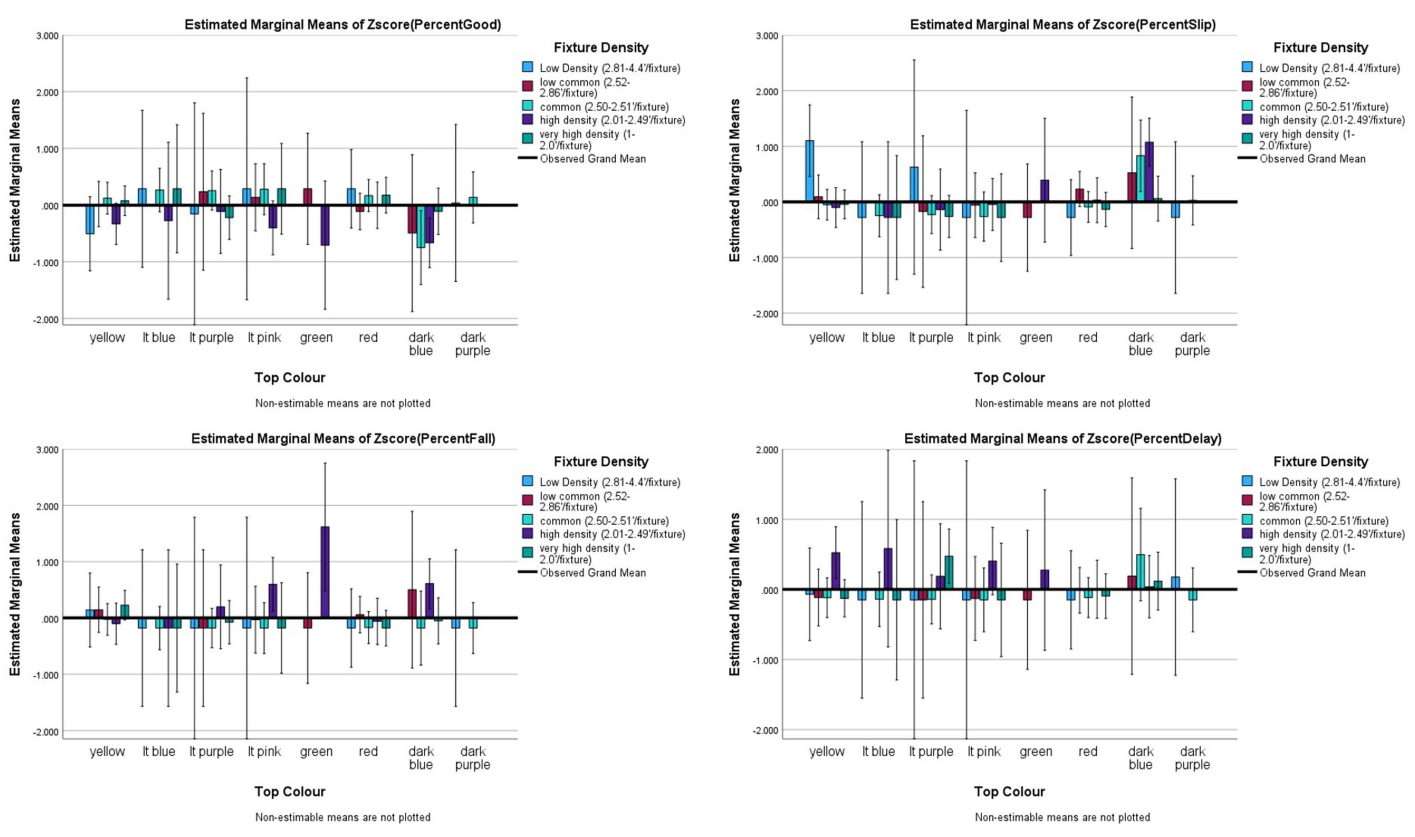
Risk factor averages for tunnel colour by fixture density with 95% confidence interval error bars included.

Assertion #3 regarded the colour of the tunnel, and perhaps the density of fixtures on the tunnel would impact risk. The data had the following representation of fixture density: “low density” (2.87–4′/fixture; *n* = 26, 4.6%), “low common density” (2.52–2.86′/fixture; *n* = 80, 14.2%), “common density” (2.50–2.51′/fixture; *n* = 201, 35.6%), “high density” (2.01–2.49′/fixture; *n* = 102, 18.1%), and “very high density” (1.50–2.00′/fixture; *n* = 155, 27.5%). The interaction of Tunnel Colour x Fixture Density was then analysed. None of the interaction effects were significant for any of the outcome variables (Good: *F*_(19,528)_ = 0.637, *p* = 0.847, *partial eta^2^* = 0.024; Slips: *F*_(19,528)_ = 1.108, *p* = 0.338, *partial eta^2^* = 0.038; Falls: *F*_(19,528)_ = 0.916, *p* = 0.563, *partial eta^2^* = 0.032; and Delayed Exits: *F*_(20,527)_ = 0.712, *p* = 0.808, *partial eta^2^* = 0.025; see Figure 6).

While none of the interactions were significant, Figure 6 shows potential issue for yellow tunnels and Slips, which appear to counter the argument regarding light and fixtures. A *post hoc* analysis was conducted specifically on the yellow tunnels, given a popular opinion that yellow is the best for lighting inside the tunnel. An ANOVA with Bonferroni post-hoc analysis for group was used to test the differences of means in the outcome variables across fixture density categories. The results indicate that low density of fixtures (1.10 ± 2.02) is significantly worse than low common (0.09 ± 1.31, *p* = 0.04), common (−0.06 ± 0.72, *p* = 0.004), high (−0.10 ± 0.52, *p* = 0.006) and very high density of fixtures (−0.03 ± 0.67, *p* = 0.005) for Slips (*F*_(4,162)_ = 3.617, *p* = 0.007, *eta^2^ = 0.082*). Common, high and very high density of fixtures did not differ significantly for Slips. There were also no differences between the yellow tunnel fixture densities for any other outcome (Good: *F*_(4,162)_ = 0.997, *p* = 0.411, *eta^2^ =* 0.024; Falls: *F*_(4,162)_ = 0.395, *p* = 0.812, *eta^2^* = 0.010; and Delayed Exits: *F*_(4,162)_ = 1.087, *p* = 0.365, *eta^2^* = 0.026).

#### Type and density of fixtures on the tunnel

Assertion #4 is about the issue of tunnel security. To address this question, several analyses were conducted. Table 1 shows the zero-order correlation of tunnel length per fixture, extra bags behind the tunnel, type of entry and body fixtures, and shape of tunnel bags.

##### Fixture types

In addition, a summary of base rates for the main types of fixtures can be seen in Table 3. A chi-square test of independence was computed to assess whether or not a tunnel is incident-free was dependent on fixture types (tunnels with cinches were collapsed into a single category due few observations of cinches with sandbags). The results indicated incident-free tunnel rates and fixtures are dependent (*χ^2^* = 54.42, *df* = 4, *p* < 0.001). In addition, a chi-square test of independence was computed to test whether the rate of incidents was independent of fixture types, and the results indicate they are dependent (*χ^2^* = 86.17, *df* = 4, *p* < 0.001).

**Table 3:** Fixtures incident frequencies and percentages.

	Fixtures			
	Total	Sandbags only	Plates & sandbags	Cinches
# of tunnel set-up conditions (rows)	563	402	95	66
# observations (total tunnel attempts)	30,418	13,324	10,371	6,723
Total # of incidents (codes: 2,3,4)	472	139	256	78
Percentage rate of incidents	1.552%	1.043%	2.468%	1.160%
# Tunnels without incidents	420	334	48	38
Percentage of tunnels without incidents	75%	83%	51%	58%
# Tunnels with slips	98	44	33	21
Percentage of tunnels with Slips	17.4%	10.9%	34.7%	31.8%
# Tunnels with falls	36	15	10	11
Percentage of tunnels with Falls	6.4%	3.7%	10.5%	16.7%
# Tunnels without delayed exits	72	33	30	9
Percentage of tunnels delayed exits	12.8%	8.2%	31.6%	13.6%

**Figure 7:**
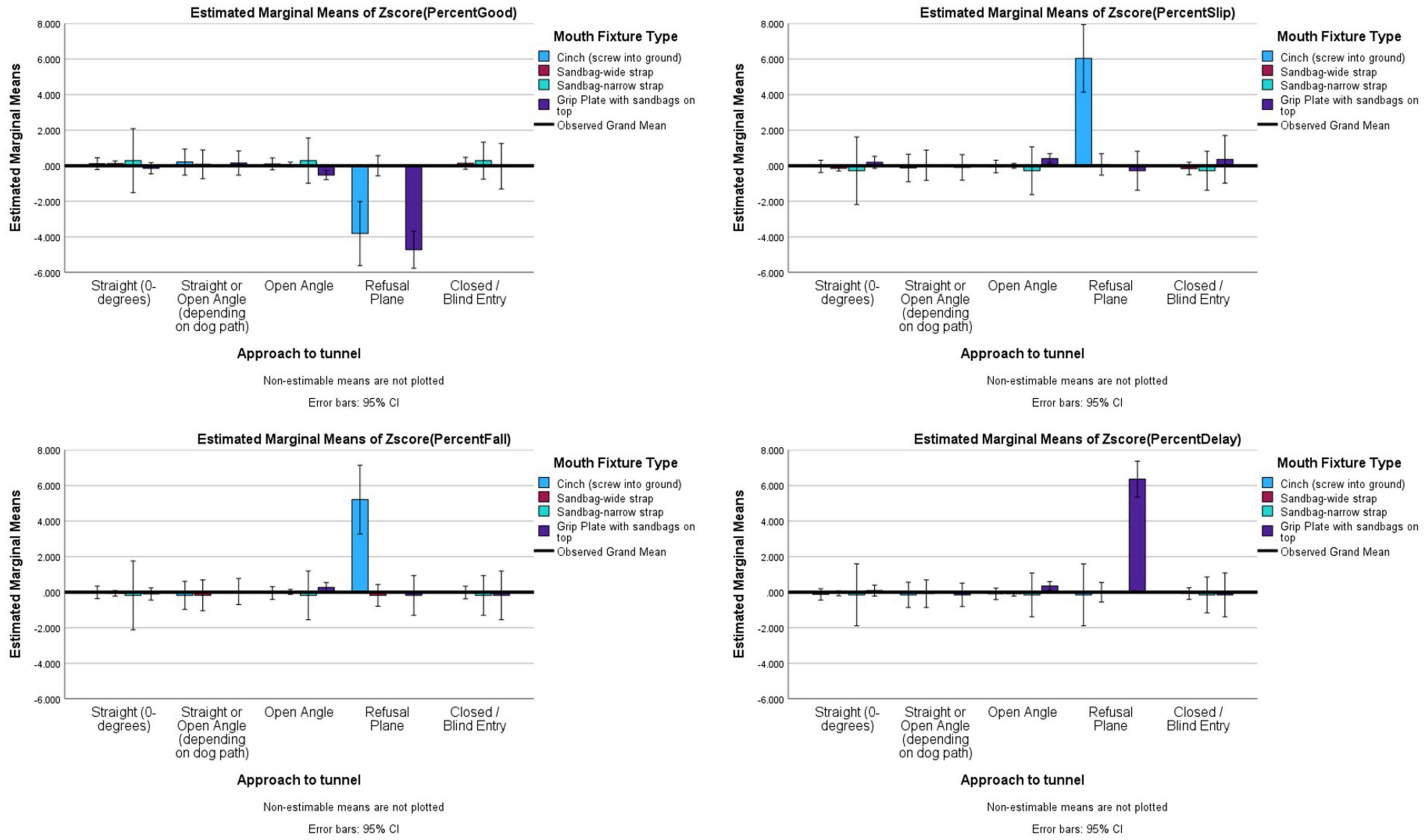
Risk factor averages for angle of approach by fixture type with 95% confidence interval error bars included.

Given fixtures act mostly to secure a tunnel from lateral forces by the dog banking, an analysis was conducted to examine the interaction of angle of approach and fixture type. All outcomes had significant interactions: Good (*F*_(9,546)_ = 6.774, *p <* 0.001, *partial eta^2^* = 0.100), Slips (*F*_*(*9,546)_ = 4.319, *p <* 0.001, *partial eta^2^* = 0.066), Falls (*F*_(9,546)_ = 3.306, *p <* 0.001, *partial eta^2^* = 0.052), and Delayed Exits (*F*_(9,546)_ = 12.192, *p <* 0.001, *partial eta^2^* = 0.167) (see Figure 7 for illustrations). For straight and blind approaches, there were no significant differences for fixture types (*p* > 0.01). For open-angle approaches, plates had fewer Good performances and more Delayed Exits than wide-strap tunnel bags (*p* < 0.01). For refusal plane approaches, cinches had more Slips (*p* < 0.001) and more Falls (*p* < 0.001) than either wide-strap tunnel bags or plates.

**Figure 8:**
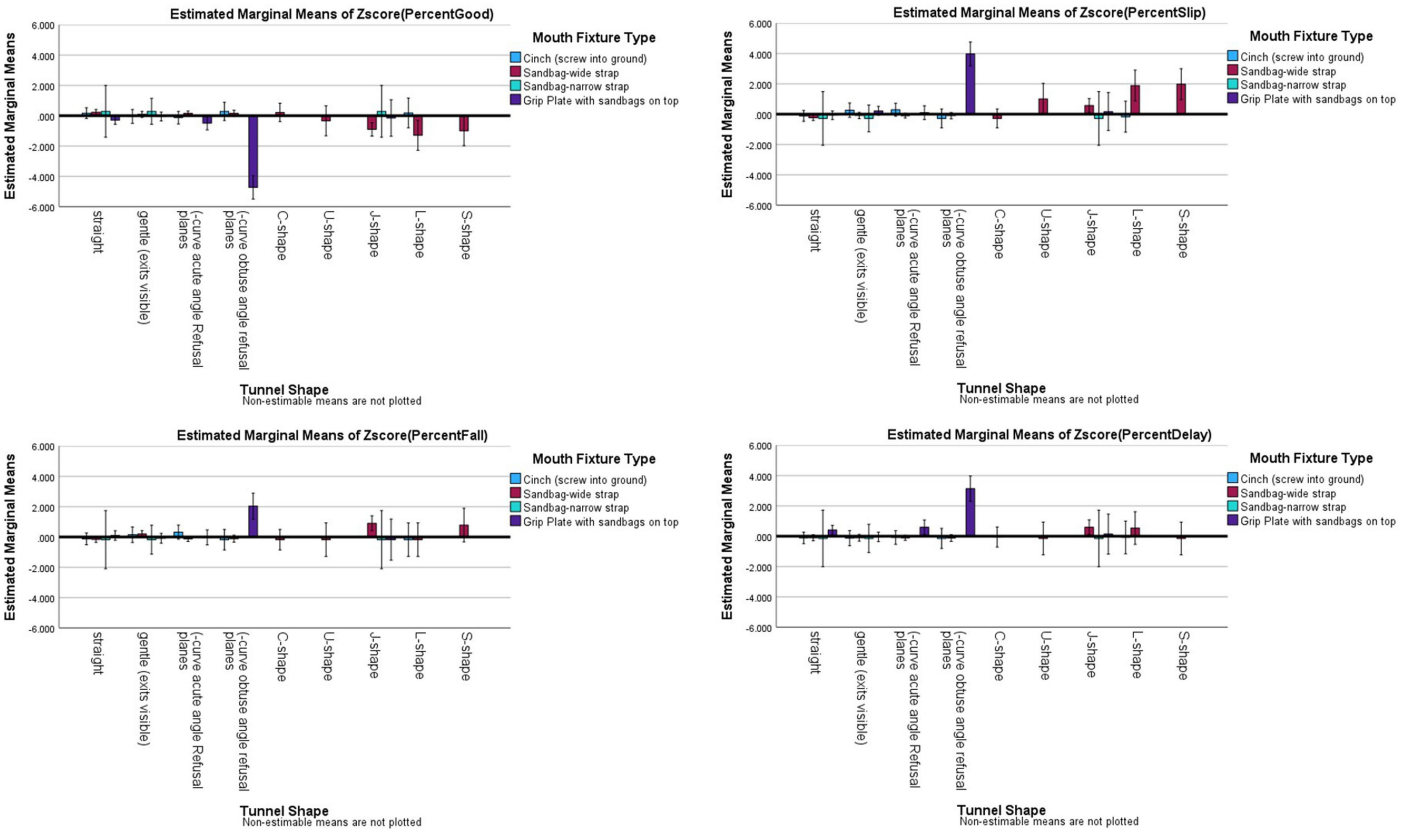
Risk factor averages for tunnel shape by fixture type on the mouth of the Tunnels with 95% confidence interval error bars included.

Given the previous findings about different shapes of tunnels, and the qualitative observations of different effects with different shapes, we examined Tunnel Shape x Mouth Fixture analysis. The results showed that there were not only main effects for tunnel shape and for fixtures on the mouths of the tunnels, but all of the interaction effects were significant: Good (*R^2^_adj_ =* 0.243, *F*_(10,541)_ = 13.398, *p <* 0.001, *partial eta^2^* = 0.198), Slips (*R^2^_adj_ =* 0.190, *F*_*(10*,541)_ = 9.615, *p <* 0.001, *partial eta^2^* = 0.151), Falls (*R^2^_adj_ =* 0.048, *F*_(10,541)_ = 3.077, *p <* 0.001, *partial eta^2^* = 0.054), and Delayed Exits (*R^2^_adj_ =* 0.095, *F*_(10,541)_ = 4.784, *p <* 0.001, *partial eta^2^* = 0.081). The interaction effects show the following. Straight, gentle curved, J-shaped and L-shaped tunnels had no significant differences between fixtures (*p* > 0.006). The (−acute tunnels with plates had significant less Good outcomes than wide-strap tunnel bags (*p* < 0.001), and more Delayed Exits with plates than either cinches or wide-strap tunnel bags (*p* < 0.001). The (−obtuse tunnels had significantly less good performance, more Slips, Falls, and Delayed Exits with plates than any other fixture (*p* < 0.001). For C, U and S-shape tunnels, there were only wide-strap tunnel bags, so these shapes could not be tested for fixture effects (see Figure 8).

In addition, the type of fixtures for the body of the tunnel did have significant correlations with outcomes. Specifically, screw-in cinches on the body of the tunnel were positively correlated to (i.e., associated with an increased number of) Falls (*r* = 0.099, *p* < 0.05), as were water-filled bags (*r* = 0.276, *p* < 0.01). Conversely, wide-strapped sandbags on the body of the tunnel were negatively correlated with Falls (*r* = −0.099, *p* < 0.05, see Table 1). None of these were correlated with Delayed Exits.

##### Fixture density

Density of fixtures was also examined to identify if there was an ideal density. MANOVA with Bonferroni *post hoc* analysis was computed using the same density codes as above. However, density may relate to the type of fixture used, so this was tested for cinched, plated, and wide-strap tunnel bags separately with Bonferroni correction (*α* = 0.017). For cinched tunnels, density was not significantly related to outcomes (*p* > 0.017). For tunnels with plates, density was also not significantly related to outcomes (*p* > 0.017).

**Figure 9:**
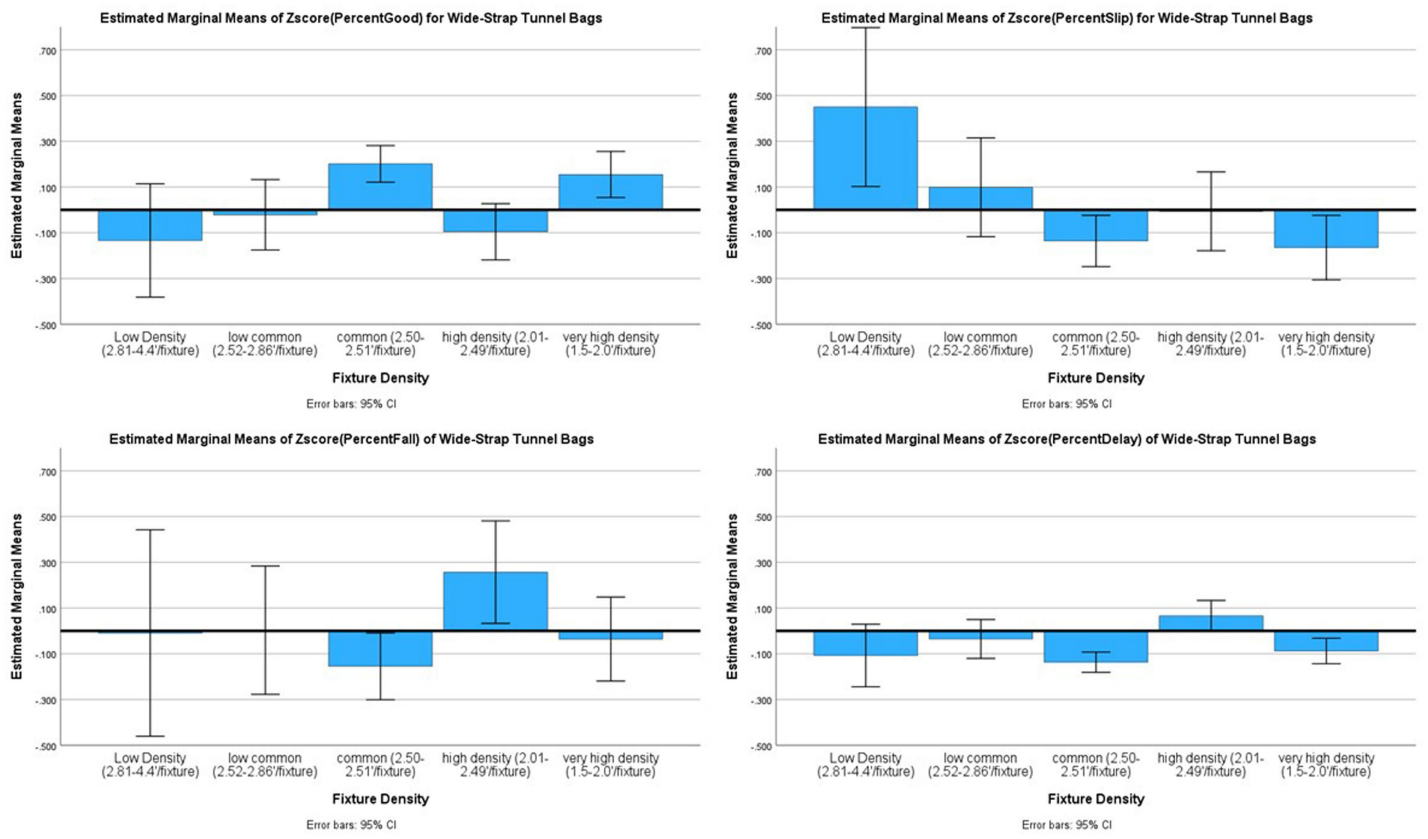
Averages for the different densities of wide-strap tunnel bag fixtures as a risk factor in dog agility competition.

For wide-strap tunnel bags, the results indicated that there were significant differences for Good (*F*_(4,391)_ = 5.746, *p <* 0.001, *eta^2^ =* 0.056), Slips (*F*_(4,391)_ = 3.657, *p* = 0.006, *eta^2^ =* 0.036), and Delayed Exits (*F*_(4,391)_ = 6.321, *p <* 0.001, *eta^2^ =* 0.061), while Falls were deemed not significant (*F*_(4,391)_ = 2.312, *p =* 0.057, *eta^2^ =* 0.023). For Good outcomes, common density was significantly better than high density (*p* < 0.017). For Slips, low density had more incidents than very high density (*p* < 0.017). For Delayed Exits, high density had more incidents than common or very high density (*p <* 0.017) (see Figure 9).

##### Vertices

Another option for tunnel security can be defined by it shifting its shape, and developing vertices. Vertices are significantly correlated and were significant predictors in the simple regression with all four outcomes, such that their presence is associated with higher rates of Slips, Falls and Delayed Exits, and fewer Good outcomes (see Table 1). There are several correlates to the existence or development of vertices in tunnels. The following are positive correlates (associated with an increase probability of a vertex existing): length of tunnel (longer tunnels have higher risk: *r* = 0.18, *p* < 0.01), shape of tunnel (i.e., U- (*r* = 0.25, *p* < 0.01), J- (*r* = 0.59, *p* < 0.01), L- (*r* = 0.23, *p* < 0.01), and S-shaped (*r* = 0.34, *p* < 0.01) tunnels), number of fixtures (more fixtures have a higher risk of a vertex: *r* = 0.12, *p* < 0.01), use of sandbags with narrow straps on the body (*r* = 0.15, *p* < 0.01), saddlebags style of sandbags (*r* = 0.13, *p* < 0.01), artificial turf (*r* = 0.13, *p* < 0.01), and dogs having the same lead as the curve on approach to the tunnel curve (*r* = 0.14, *p* < 0.01). All of these were regressed against the three outcome variables (entry vertex, middle vertex, and exit vertex). The results indicated for entry (*F*_(23,492)_ = 28.568, *p* < 0.001, *R^2^_adj_* = 0.552) and exit (*F*_(23,492)_ = 20.407, *p* < 0.001, *R^2^_adj_* = 0.464) vertices, the only significant predictors were shape of the tunnel (J- and S-shaped). However, the middle vertex (*F*_(23,492)_ = 24.360, *p* < 0.001, *R^2^_adj_* = 0.511) was predicted by tunnel shape (U- and L-shape), but also by the number of bags (more fixtures: *F*_(11,492)_ = 2.505, *p* = 0.005, *partial eta^2^ =* 0.053) and artificial turf (*F*_(1,492)_ = 102.51, *p* < 0.001, *partial eta^2^ =* 0.020).

##### Wire pitch

**Figure 10:**
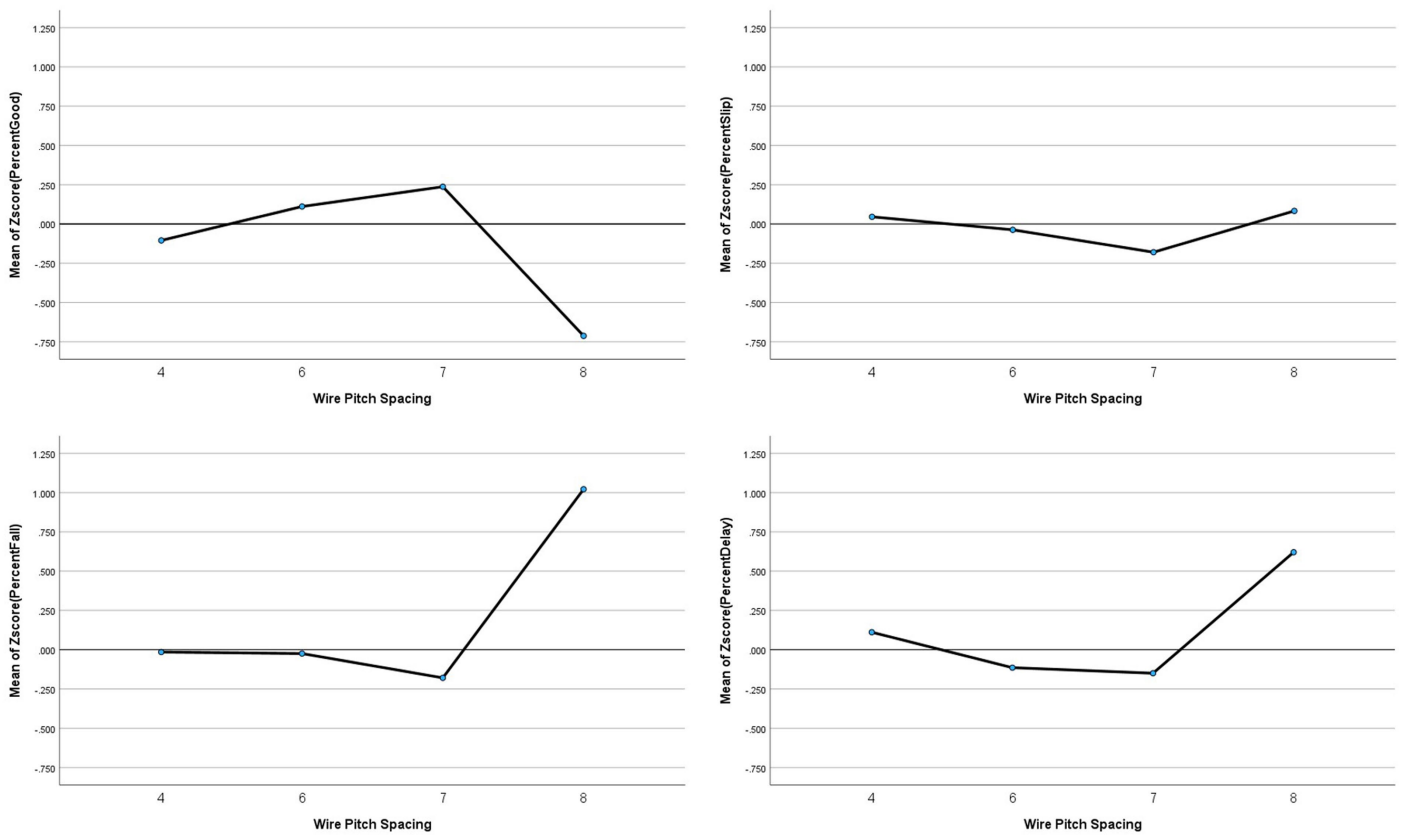
Averages for the different wire pitch spacing (in inches) as a risk factor in dog agility competition.

Another variable that may come into play with the shifting shape of the tunnel is the wire pitch (4″, 6″, 7″, 8″) as more densely laid wire (e.g., 4″ pitch) may provide more stability to the tunnel. To test if this had an impact on outcomes, a one-way ANOVA was conducted to test group differences on this factor. The results show that there is a significant difference for all outcomes except for Slips (Good: *F*_(3,542)_ = 3.955, *p* = 0.008; Falls: *F*_(3,542)_ = 3.892, *p* = 0.009; Delayed Exits: *F*_(3,542)_ = 3.650, *p =* 0.013). Post-hoc analyses using Bonferroni correction indicated the 8″ wire pitch (20 cm) had fewer Good performances than 6″ (15 cm) pitch, and 8″ wire pitch had more Falls than the 4″ and 6″ pitches (*p* < 0.05). For Delayed Exits, there were no simple group differences (*p >* 0.05) which means there is a difference between one and a cluster or between clusters (see Figure 10).

#### The angle of approach to the tunnel

**Figure 11:**
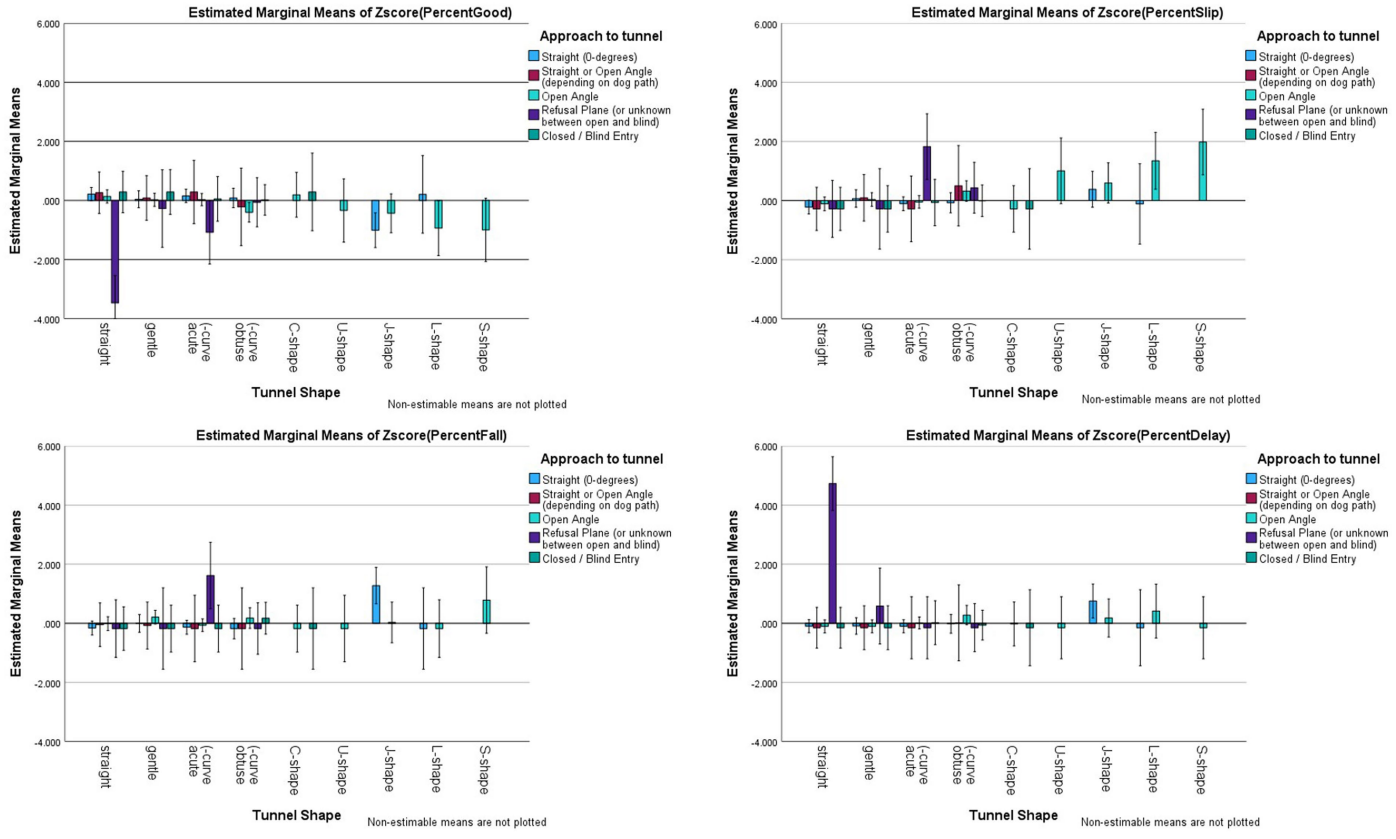
Risk factor averages for tunnel shape by angle of approach with 95% confidence interval error bars included.

Assertion #5 was tested by examining the impact of the angle of approach, and the shape of the tunnel on outcomes. To assess this the Tunnel Shape x Angle of Approach interaction was tested. There were significant interaction effects (Good: *F*_(15,535)_ = 2.900, *p <* 0.001, *partial eta^2^* = 0.075; Delayed Exits: *F*_(15,535)_ = 5.447, *p <* 0.001; *partial eta^2^ =* 0.132); however, Slips (*F*_(15,535)_ = 0.969, *p* = 0.487, *partial eta^2^ =* 0.026) and Falls (*F*_(15,532)_ = 1.285, *p* = 0.206, *partial eta^2^ =* 0.35) did not have a significant interaction between tunnel shape and angle of approach (see Figure 11 for illustration). For two outcomes (Good, Delayed Exit), it was one single combination that had significantly worse outcomes than the other angles of approach: straight tunnels with refusal plane approaches or where the *handler’s or dog’s choice of path* could make it an extreme open-entry approach or blind approach. In other words, these approach angles varied between ~75-105-degrees and it was up to the handler’s choice (or dog’s performance) on the obstacle prior to the tunnel that defined the actual approach (see Supplemental Materials Figure B for approach ranges).

It should be noted that certain combinations were not significantly different from other combinations, but were greater than the grand mean for Slips, Falls, or Delayed Exits. In particular, open angled approaches for L- or S-Shaped tunnels (in fact, the coder noted the S-shaped was caused by the open-angled approach and insufficient weight on the sandbags that created the S-shape), had more slips/missteps than the grand mean. Similarly, refusal plane approaches on (−acute tunnels were also greater than the grand mean for Slips. For Falls, refusal plane approaches on (−acute tunnels and straight approaches to J-shaped tunnels were greater than the grand mean. For Delays, straight approaches to J-shaped tunnels had greater delays than the grand mean.

## Discussion

This research set out to address the question: *Which tunnel, design, environmental, experiential factors (if any at all) have a predictive relationship to injury-risk incidents?* Initial analyses of first order correlations highlight that outcomes of incident free performance (*Good*), visible slips or missteps (*Slips*), visible shoulder or hip contacts or falls (*Falls*), and unseen incidents or discernable delayed exits (*Delayed Exits*) are not solely related to one factor. Rather, competition level, type of ground and conditions, design components (shape and approach), and tunnel characteristics may play a role.

The simple regression identified four factors for incident free (*Good*) performance, three of which were associated with lower rates (middle vertex, exit vertex, and refusal plane approaches) and one was associated with greater frequency of incident free performances (U-shaped tunnel). Five factors for visible slips/missteps were identified, three of which associated with increased risks: red colour (−obtuse, L-shape and vertex at the exit, and one associated with reduced risks: dark purple tunnel). For visible shoulder or hip contact or falls, the simple regression did not aid in identifying factors. For a dog to lose its balance and ability to remain upright appears to be a more significant issue for competing agility dogs. Thus, Falls appear to not be due to single factors on their own, and requires the examination of interaction effects. Delayed exits/unseen incidents were found to be predicted by four factors in the simple regression: U- and L-shape reduced risks of delayed exits; whereas, vertex in the middle and refusal plane approaches were associated with increased risks of delayed exits.

### Tunnel shapes and the dog path: U, L and spiral curve

Given the surprising results for the U-shape and the L-shaped tunnels, the data was re-examined for these shapes to see if there were any clues to these results. There was one U-shaped tunnel, which was 20′ with 8 sets of wide-strapped sandbags on grass at an international level event. The tunnel was coded for three different ground conditions due to changing conditions during the course. A notable thing about this tunnel was it mirrored the dog’s path on the ground fairly closely.

The L-shaped tunnels comprised of six tunnels in the data, and were mostly 20′ (one was 15′), half were fixed with wide-strap sandbags, half were fixed with cinches. All but two had middle vertices (hard bends) in them. The two without vertices had zero delayed exits and one slip (out of 283 performances). These two tunnels may have met a “spiral curve” build more so than a true L-shape given the lack of a vertex (a spiral curve, also known as transition curves or clothoids, is a curve with a varying radius to aid in transitioning into and out of a curve. This design is used in road construction to prevent slipping and collisions, and helps counteract centrifugal forces with banked edges (like the wall of the tunnel). Of the four tunnels with vertices, half of them had delayed exits with an incident rate of 2.6%, and the other two only had 66 performances. Thus, L-shaped tunnels with vertices appear to perform differently for delayed exits than L-shaped without a hard bend (i.e., spiral curve), and vertices should be avoided when building and running a course.

### Vertices

Similarly, the relationships between the shape of the tunnel and its relationship with the presence of vertices needs to be examined further. In our data set, J-shaped and L-shaped tunnels were defined with a vertex to determine where the curve was located (entry vs. exit for J’s, and middle for L’s). However, coders might not have distinguished between location of a spiral curve (as found with a J or L-like spiral curve) versus a hard-angled curve that develops (like a harsh L angle, or a vertex on any shape of tunnel due to the extending of the exterior wall from forces applied by dogs banking). Thus, future research needs to examine if L and J shaped tunnels are inherently risky or if it is due to a sharp angled vertex on the outside wall that is the issue. Theoretically, we expect it is the sharp angle vertex, not a curve per se (e.g., spiral curve) that is problematic.

### Common safety assertions vs. statistical results

#### Level of traction inside tunnels

Beyond the simple, first-order and regression results, this study examined in more detail five themes identified from general dialogue. First the level of traction is relevant for incident rates. Half anti-slip tunnels were correlated with higher rates of Falls, and when conditions were damp, no anti-slip tunnels were predictive of more Slips and fewer Good outcomes than full anti-slip. Thus, this study supports the assertion that full anti-slip grip tunnels aid in reducing incident rates with tunnels. However, using full anti-slip tunnels is not a cure-all.

The results of this study also confirm that damp grass is associated with increased risks compared to dry (not burned) grass, such that this condition was associated with more falls. In addition, dry (not burned) grass outperformed dry sand for Good and Slips, and dry grass outperformed dry artificial turf for Slips. Thus, this research confirms traction is a risk factor for Slips, Falls, and Delayed Exits. The best performances occurred where tunnels had full grip and where no environmental “lubricants” (like water) transferred into the tunnel on grass or sand.

Within this study, all of the artificial turf (brands and types varied, but all were deemed appropriate for agility competitions) venues were indoors and were considered dry. Despite the turf being dry and not transferring “lubricants” into the tunnels (none had infill), the artificial turf was associated with a higher rate of Slips, falls and delayed exits than dry grass. One of the attractions of artificial turf for agility is the improved traction on the ground for the dogs. One of the potential benefits of improved traction on ground may be higher ground speeds achieved by the dog. This higher speed may be associated with less ability to control their balance or more significant impact with tunnels when the tunnel alters their trajectory, even with full anti-slip tunnels (there were no half anti-slip or no-anti-slip tunnels on artificial turf within this data).

#### Length and shape of tunnels

Regarding the length and shapes of the tunnels, the results provided some new information. First, the results suggest that the longer the tunnel, the higher the risk for Slips and Delayed Exits (and fewer Good performances). The tunnels with the best outcomes were the shortest tunnels (10′/3 m, 13′/4 m), and their shapes did not have to be completely straight. For the 10′/3 m tunnels, very gentle curves (where the exit was still visible through the tunnel) were equally safe in terms of Slips, Falls, Delayed Exits (0 incidents, secured with wide-strap sandbags). As noted earlier, there were a total of 8 incidents (0.43% incident rate) for the 10′/3 m tunnels, all of which were straight, but fully secured with cinches. All other 10′/3 m tunnels, that were secured with wide strap sandbags, had zero incidents *with or without* gentle curves in them (0.00% incident rate out of 1,178 performances). The 13′/4 m tunnel that had one Fall and one Delayed Exit was straight (under very wet conditions with a full anti-slip surface), and one that had an (−acute refusal plane shape had one Slip. All others were straight or with gentle curves and zero incidents. Thus, the argument that 10′/3 m or 13′/4 m tunnels must be straight is not supported by this evidence. Rather, provided they are secured with wide-strap sandbags, and the exit is visible from the entry, these tunnels appear to have little to no risk.

Furthermore, the C-shaped tunnels were not statistically significant predictors for incident rates. It should be noted that it was qualitatively observed that these shapes supported the dog’s trajectory path that they were already on, or heading onto (curved paths on course) and did not tend to be used with fast-straight lines on approach. Furthermore, the most common tunnel shape now, the (−shaped, was associated with the most incidents, when its refusal planes created obtuse angles for 16′/5 m tunnels. This shape for 16′/5 m tunnels had significantly higher risk for Slips compared to all other shapes of 16′/5 m tunnels (including J-shaped), and higher risk for Falls compared to less curved 5 m tunnels: straight, gentle, (−acute angle refusal plane shapes).

The results do support the concern of S-shaped and L-shaped tunnels. It should be noted that while the organization and course build did not have an S-shape, the tunnel morphed into an S-shape, likely due to insufficient securing and angle of approach into the tunnel. The S-shape results may be associated to insufficient weight in the tunnel bags, or due to the type of tunnel bags (saddle bags). L-shaped tunnels did *not* have the issue of insufficient securing, and were problematic for dogs when they had a vertex in them (e.g., an abrupt turn).

#### Colour of the tunnel: visibility

The third assertions were that tunnel colour and lighting inside the tunnels impacts tunnel safety. This assertion had no support from the data, except for red tunnels (which were positively associated with Slips in the regression and first order correlation), but there were also results that countered it (dark purple tunnels were associated with fewer Slips). Examining colour more closely within the data, there was one international event which exclusively used red tunnels, and the conditions for the event were extremely wet. This may have resulted in spurious results regarding the colour red and Slips. On the whole, darker colours did not have higher rates. Future research should examine the red/green colour spectrum for tunnel incident rates further. Moreover, having more bags on a tunnel did not have an impact on incident rates; whereas, insufficient fixtures on the yellow tunnels was associated with more Slips.

These results regarding tunnel colour and Slips, Falls and Delayed Exits do not include refusals. It is quite likely that handlers, judges, coaches prefer lighter tunnels as there may be fewer refusals (a performance issue, not a safety issue). Qualitatively, two dogs were seen to refuse a dark tunnel at a night trial, and one dog refused a red tunnel at a daytime trial, but as they did not perform that tunnel, there was no data included for those three dogs on those particular tunnels. Thus, judges, trainers and handlers may still show a preference for lighter colours other than red, dark blue, purple, or black due to improved performance that is judged.

#### Type and density of fixtures on the tunnel

##### Fixture types

The fourth factor was the type and number of fixtures on the tunnel. Here the results showed some interesting issues. First, the results of the chi-squared test of independence indicate that tunnels with plates or cinches have fewer than expected incident-free tunnel rates, given the rate for all types. In addition, it indicates tunnels with plates on the mouths have higher incident rates overall than what would be expected (see Table 3).

The results suggest tunnels that were exclusively affixed with wide-strap sandbags filled with sand/pea gravel (not water), had the best performance outcomes in terms of incidents. It should be noted that not all sandbags performed equally well; narrow-strapped bags on the body were associated with more Slips. Cinches also fared well in terms of overall incident rates; however, a higher proportion of cinched tunnels had incidents (just fewer per tunnel). As noted earlier, the only incidents that occurred with 10′/3 m tunnels were ones that were fully affixed by cinches. Some tunnels secured with cinches only appeared to wobble up top, while staying stationary in the location on the ground. This wobble appears to be created by the wire pitch adjusting to forces from the dog while banking on the tunnel. Without counterforce (like the ground or sandbags), the inside perimeter of the tunnel’s body morphs with each footfall. This wobble action may be a contributor to the Slips and Falls observed due to loss of balance, particularly for large dogs. Future research needs to examine this further.

The plates were problematic both in terms of rates of incident-free tunnels and in terms of overall incident rates. Specifically, plates were problematic for (−obtuse shaped tunnels for visible slips/missteps, visible shoulder or hip contact/falls and delayed exits/unseen incidents. Additionally, when dogs entered the tunnel from an open-angle approach, there were significantly more Delayed Exits. Qualitatively, it was observed that the dogs (regardless of size) who banked the entry, got one to two more strides into the tunnel before significantly losing flow (as evidenced by tunnel wall movements and following of the dog’s path/speed). It is uncertain if the delayed exits were due to unseen slips, falls, or due to a loss of balance which necessitated un-cued collection.

In addition to the mid-tunnel delays, the tunnels with plates also presented significant issues that required further discussion within the research team on types of coding for outcomes. Specifically, large fast dogs were seen to get “hung-up” on the exit of the tunnels that were redirecting their path to lock onto the approach of the next obstacle (e.g., dog walk). These incidents occurred with the dog having a relatively straight approach, and a straight exit from the tunnel, but the tunnel was curved in the body (without vertices). This was coded as a Slip (slip/misstep) if the dog remained fairly upright while getting hung-up on the exit. It was coded as a Fall if the dog experienced a substantial contact with the side of their body (e.g., shoulder or hip) on the wall of the tunnel while no longer maintaining an upright position. In addition, smaller dogs appeared to have unexpected trajectories out of these tunnels. This effect would be a problem for judges when designing and expecting dogs to have a specific trajectory on exit, especially for obstacles that require a “locked-on” trajectory is needed for safety (such as contact obstacles, tire jump).

At one local-level event, the judge noticed this issue with the plates and removed them, and the “hang-up” incidents stopped. At a national-level event, two coders inspected the tunnels when a spike of delayed exits were noticed in data collection. It appeared as though there were some subtle vertices developing in the middle when the rest of the tunnel moved (despite high density of tunnel bags with sufficient pebble/sand) without the exits moving at all. Alternatively, it is possible that the dog’s body takes more force due to the solid (no transfer of lateral force vectors – e.g., no “give”) entry wall. Regardless of the underlying mechanics, these results call for more research and significant caution in the use of these fixtures.

The plates did not appear to have an effect on Delayed Exits, Slips or Falls when there were straight or blind-entry approaches and when the dog did not contact the walls of the tunnel. However, in this type of tunnel performance, the additional security to the ground is unnecessary as there is little to no threat of the tunnel shifting without banking.

Future research is also required to examine the effects of fixtures on the trajectories of the dogs *exiting* the tunnel. If some fixtures are significantly altering dog trajectories or balance on their path to equipment that requires balanced dogs with predictable trajectories, then future research needs to examine this in more detail. Specifically, the qualitative assessments from some of the high-level events suggested there were trajectory and balance issues for tunnels with plates and cinches, but not for wide-strap sandbags. In particular, it was noticed that with the same sequence set-up (relatively straight approach, curved tunnel to set the path onto the dogwalk), the tunnel mouth would shift laterally along the ground a bit while the dog’s trajectory continued forward in the expected manner and in balance with wide-strap tunnel bags; however, either the dog would collapse into the tunnel wall with plates or bounce slightly off the expected path with cinches (or plates for small dogs). It appears that the wide-strap sandbags convert the angled force vector into lateral force (onto the tunnel mouth) and forward force (dog) vectors, leaving the dog in balance and on the expected, designed trajectory. The cinches absorb lateral force somewhat (via the wire pitch), but may act as a spring and bounce the dog back more than sandbags shifting away. Conversely, the tunnels with plates and sandbags do not convert angled force vectors off the dog, and the dog appears to absorb the full force into their body, thus appearing to get hung-up. Thus, the types of fixtures need to be studied to ensure they are not creating unpredicted exits that then impact the safety of the next obstacle, like the dogwalk.

The data in this study cannot provide insight on the weight for the sandbags. The most common version used in the data set has manufacturer recommendations of ~35 lb. (15-16 kg) per bag. Qualitative observations noted one show had bags notably lighter than that, and those tunnels were morphing in shape with open angle entry approaches and had higher rates of slips and falls. Future research needs to confirm ideal weight.

##### Fixture density

The results of this research also suggest there is a “better” and a “riskier” density of wide-strap tunnel bag fixtures, specifically the common density of 2.50–2.51′ per fixture had the best outcomes for Good performances, Falls and Delayed Exits. Having a high density of wide-strap tunnel bags (2.01–2.49′/fixture) was associated with fewer Good performances and more Delayed Exits. Having low density of wide-strap tunnel bags (2.81–4.4′/fixture) was associated with more Slips. In addition, when considered with colour, yellow tunnels with low density of fixtures had fewer Good performances. This means the optimal fixture density (2.50–2.51′/fixture) is the following: 4 wide-strapped sandbags for a 10′/3 m tunnel, and 8 wide-strapped sandbags for a 20′/6 m tunnel. Optimal density does not compute for the 13′/4 m and 16′/5 m lengths, thus to avoid the higher risk density, the 13′/4 m should have 5 (not 6 which is high density) and the 16′/5 m tunnel should use 6 (not 7 which is high density) wide-strap sandbags. It should be noted that incident rates for cinches and plates were not significantly related to density.

##### Wire pitch

Related to stability is the wire pitch. The results show that the best performing pitch overall is the 6″ pitch, the worst is 8″ pitch. The 4″ pitch performs equally well to the 6″ pitch for Slips and Falls, but 4″ and 8″ pitch have more risk for Delayed Exits when compared to 6 and 7″ pitch. A potential explanation for this is that with new 4″ pitch tunnels, getting full expansion of the tunnel may be more difficult during course building, and this very slight slack may lead to trips on fabric or the body of the tunnel shifting during the class due to dogs impacting the walls in the middle of the tunnel and create vertices for other dogs in the same or subsequent class. It should be noted, that during some trials, the tunnels were left alone and nested throughout the full day or used in very large classes without being reset in the body. The tunnel bodies did morph throughout the day and vertices could be found.

#### The angle of approach to the tunnel

##### Tunnel shape and angles

The course design and approach to the tunnel was confirmed to be an issue. Specifically, the approach that surrounds the refusal plane is the highest risk approach when the tunnel is (−shape. Blind approaches and open-angled approaches are not problematic, but that zone where either handler-choice or dog choice switches from blind to open-angle (or vice versa) or judge design places the approach on the refusal plane, is a high risk for Slips and Falls.

Most importantly within the course design appeared to be the shape of the tunnel *relative to the dog’s path on approach.* Tunnels that supported the pre-existing trajectory of the dog (either straight or curved) tended to have excellent outcomes (no incidents). However, when the tunnels, either by design or by build, suddenly introduced a change of trajectory, the effects were seen in slips/missteps, shoulder contacts/falls and delayed exits/unseen incidents. The sudden change of trajectory could be seen when a dog entered the tunnel along the refusal plane or extreme open angle approach (i.e., without prior collection) and the tunnel was uniform in curve that did not match their trajectory. Examples of this include: a (−shape with an open-angle approach, a straight approach into an L-shaped tunnel that has a vertex, or whenever there were clear, sharp vertices within the tunnel.

These results, along with dog path and tunnel shapes, were shared with an Engineering professor (PhD) who explained this is similar to road construction and car crashes. When drivers face a turn in the road, abrupt turns (with speed) result in high rates of crashes. Similarly, immediately entering a constant curve, like the (−shape, requires drivers to immediately assume the correct turn and hold it constantly to avoid collision. The best design is a spiral curve where the curve gradually changes from current trajectory into a turn, then out of the turn, without creating any sudden changes (see Supplemental Materials Figure C for illustration). This theory, as it might apply to tunnels, was confirmed by an independent Engineer who is active in agility in another country. Thus, while there is a push for (−shaped tunnels with constant curvature throughout the tunnel as a way to avoid C-, U-, J-shaped or L-shaped; this research and the engineering theories behind controlling changes of direction with speed, suggest this may not be the best approach. More research needs to test the spiral curve versus (−shape versus C-shape with respect to dog paths and speed on approach to tunnels.

##### Other course design factors

Expected leads on approach had no bearing on incident rates when other factors were considered at the same time. The location of tunnels within the course (i.e., obstacle number) had no impact on incident rates. Event level was not relevant; however, class level was correlated to Delayed Exits (higher levels had more Delayed Exits). This finding is contrary to expectation that the more novice dogs may stop to sniff. These delayed exits were especially seen at high level classes with fast, well-trained dogs on new equipment. This suggests it is an interaction with the equipment and not an issue with training or other environmental factors like scent, food or foreign object.

### Comparison to previous research findings

When we compare the results of this study to previous research on agility injuries and the tunnel survey in the UK (24), we see some important synergies. Our results confirm that full anti-slip tunnels have fewer falls than half anti-slip when damp, and full anti-slip tunnels have fewer slips and more good outcomes than no anti-slip tunnels in general. Lott’s (24) study noted full anti-slip was better than no anti-slip and half anti-slip overall. Our study highlights under what conditions they are better. Similarly, our study replicates the results of the (−shape tunnel having issues with slips, falls, delayed exits, like Lott’s (24) study, and that straight and gentle curves are better. However, our study highlights that the angle of approach and types of fixtures play a role in how these shapes predict slips, falls and delayed exits. Our research did not find support for “dark tunnel colours” predicting incident rates, with the exception of red tunnels. In fact, dark purple predicted better outcomes. However, our study explicitly excluded refusals; whereas, responders to Lott’s survey may have included it, which may introduced confounding between performance and safety.

Ring surface and wet vs. dry conditions (24) were found to be relevant, and that there was an interaction effect. Levy et al. (5) found dry outdoor conditions predicted injuries in agility; however, it is unclear if it was dry sand, burnt dry grass, or dry green grass. Our results indicate dry grass had the fewest tunnel incidents, and dry indoor turf had the highest tunnel incident rates. Level of competition risks for injury (3, 21) were relevant for this study such that level of class was correlated with Delayed Exits; however, it was not a predictive factor when all other factors were considered in the regression.

### Limitations

The limitations associated with this research are the following. First limitation is there were some factors that were not captured, either due to inability to accurately measure or missed in the design. More specific environmental factors, such as temperature, humidity levels, wind speed were not recorded. It is possible that temperature and humidity levels could interplay with ground conditions and the performance of the anti-slip grip or tunnel temperature (particularly for the darker coloured tunnels that may retain more heat from the sun). Future research may want to examine this.

Second, it is possible some tunnels at some world level events had plates that were not coded. Of these events, 56 tunnel cases (2,868 observations, 36 incidents, 1.26% incident rate) were confirmed as not having plates due to close-up review of video. Another 62 world-level tunnel cases (2,510 observations, 27 incidents, 1.08% incident rate) could not be confirmed due to live feed and inability to zoom into view. Given the similar rate of incidents, it is assumed these events did not have plates.

Third, the weight of the fixtures (sandbags) was not measured due to inability to accurately assess that as observers. Future research should examine what is the optimal weight in the wide-strap sandbags (pebble). Is there such a thing as too heavy for sandbags (aside from ergonomics and course builder safety)? At what point is it too light? Given the variety of sizes and shapes of tunnel bags, should there be a weight per meter of tunnel length as opposed to weight per bag? In addition, are there designs for the bag’s traction with the ground and the bag’s traction with the walls of the tunnel that lead to better or worse outcomes? There are a variety of designs and materials on the market that should be tested.

A fourth limitation is the lack of variance of age of the tunnels. Age details were not available for the half anti-slip tunnels, and the oldest tunnels in the data were 5 and 6 years of age, all of which were no anti-slip tunnels. (The no anti-slip tunnels ranged in age from 3 to 6 years of age, with an average age of 5.05 years.) The vast majority of the full anti-slip tunnels in the study were new, with ages ranging from new (0) to 2 years old, with an average of 0.31 years. Future research needs to examine what is the lifespan of the internal grip coating in the tunnels, and the results of this research cannot be generalized to full anti-slip tunnels that are over 2 years old or no anti-slip tunnels over 6 years of age.

In addition, the exit paths (straight, loose turn, tight turn with same lead, tight turn opposite lead) in the course design (dog’s path) were not measured. Qualitatively, there were some observations that a J-shaped tunnel with the vertex at the exit actually supported some dogs on their tight turn exits, as did some C-shaped tunnels. Similarly, the ground conditions impacted some slips outside the tunnel (this was not coded as a slip as it was before the entry or past the exit of the tunnel). Future research should consider risks of slips and falls on approach and after the tunnel, not just while the dog is partially to fully inside the tunnel.

Related to this is that this research did not examine how tunnels created conditions for subsequent obstacle performance and safety on the ground. Qualitatively, it was observed that gentle curved to (−shaped tunnels with straight entries could result in high speeds reached by the dogs. One course, in particular, had two back-to-back like this, and the dogs needed to make a loose 180-degree turn for a jump. The wet (active rain) conditions on grass resulted in slips, a fall, and near collision with handlers 2–3 strides past the second tunnel. Future research needs to examine how tunnels are creating speed and how that interacts with conditions for balance (e.g., dogwalk, jumping) and ability to safely perform the next portion of the course (e.g., turns on the ground). With a growing trend of two to three back-to-back tunnels on course, this concern is relevant for safety, particularly if speed increases risks of incidents.

This data set also did not include any tunnels with plates on the body of the tunnel, they were only present at the tunnel mouths. However, with the effects seen within this study, we would expect to see more Delayed Exits due to a lack of transfer of lateral force vectors onto the dog’s body with plates present along the full length of the tunnel. Our data represent events that occurred across several countries, agility organizations and equipment manufacturers, as previously described. However, data did not include all equipment and organizations that exist globally. While there was a variety of tunnel vendors, styles of fixtures, and age of tunnels within this data, some countries may have different vendors and equipment attributes than those examined in this study.

Additionally, the definition of “open-angled approach” had a large range of angles. It included ~30-degrees to ~80-degrees on approach. The spike of incidents for (−shaped tunnels was when the dog’s path had options between open-angled and blind approach. This suggests that angles around 90-degrees have significant risks; however, this does not imply 60 to 80-degree approaches are safe. Most open-angled approaches ranged 30-45-degrees. Future research is needed to identify safety for more nuanced angles. In addition, this research suggests C-shaped did not have significant risks; however, this might not hold true if there are fast lines with straight entry into that shape.

A factor that was not included was the length of the dog’s training and their experience level. However, our research did include all levels of competition from entry-level local trials to national and team tryouts to international events. Each level can be considered a proxy variable for the amount of training and experience of the dog. If dog experience is a factor as has been shown elsewhere [e.g., (2)], the lowest levels of classes/events would have the worst rates; however, that was not the case in this research.

### Implications

Given, the overall incident rate was 1.55%, judges and competitors may use this as a standard to identify tunnels that are having a significantly higher risk for incidents (e.g., more than 2 incidents over 100 performances). In terms of risk factors, this research suggests that the worst case scenario for tunnel safety would be: tunnels that are 8″ pitch (visible slips/missteps and visible shoulder or hip contact/falls) or 4″ pitch (delayed exits), no anti-slip or half anti-slip interior, 20′ (6 m), and possibly red, with fixtures that include plates or cinches placed at a high density (2.01–2.49′ per fixture), in a ring with artificial turf, damp grass or dry sand, and in the shape of an L with a vertex, S-shape or (−obtuse shape, and with a refusal-plane approach in the design. There were no tunnels that met that exact description, but there was a 4″ pitch (full anti-slip), 6 m, red tunnel, affixed with plates on the tunnel mouth and sandbags with wide-strap sandbags at a density of 2.2′/fixture, on artificial turf (−obtuse shape tunnel with open-angle approach, which had an incident rate of 6.25% (4 times greater than 1.55%). Conversely, the best case scenario appears to be: tunnels that are 6″ pitch, full anti-slip interior, 10′ (3 m), with either a straight or gentle curve, and any colour but possibly red, with fixtures that are wide-strap sandbags placed at 2.50–2.85′ per fixture, in a ring with dry (not burned) grass, and in the shape that supports the exist dog’s path on the ground, straight or with blind entry or gradual changes in the trajectory (spiral design). There were six tunnels that met these criteria, and there were no incidents over 185 performances (0.00%; the expected rate is 3 incidents).

This research has several implications for agility organizations, judges, coaches, competitors and equipment manufacturers. First, for organizations, it is clear some organizational policies are not substantiated by evidence (e.g., disallowing any gentle curve on 10′ (3 m) or 13′ (4 m) tunnels, push towards the (−shaped tunnel and removal of other curved shapes. The shape with the worst outcomes is the (−shape (made worse with open-plane or refusal plane approach, or with plates). Thus, organizations should examine the shape of the tunnel relative to the dog’s path on approach and on exit and support the path instead of having a general shape standard.

On the other hand, this research suggests it would be beneficial for organizations to regulate the types of fixtures, density of fixtures, wire pitch, and type of interior of tunnels. The data suggests we should promote wide-strapped sandbags (either cylinder or triangle shaped bags), give significant consideration to the suspension of plates and possibly cinches as fixtures, use a density of 2.5′/fixture, avoiding greater than 2.81′/fixture and 2.01–2.49′/fixture, and prioritize the use of 6″ pitch tunnels over 4″ and 8″ pitch tunnels, and full anti-slip tunnels, pending more research. We would also recommend ensuring a minimum weight per meter for fixtures; however, our research does not have the data to recommend the specific weight required.

For design, organizations should also regulate course design to prohibit approaches to (−shaped tunnels near the refusal plane. It would behoove judges to avoid this in their design regardless of organizational requirements. Straight, gentle curves with visible exits (for all lengths), and curved shapes were fine. The dog’s path on approach has implications. There are no implications for designing where the tunnel is in the course in terms of its obstacle number in the course.

For judges, coaches and handlers, the implications of banking of entries and the speed/force of the dog upon connecting with the tunnel had significant design implications but also handler-choice and training implications. The implication of the banking was amplified with the plates on the entries or insufficient securing of the tunnel with light tunnel bags. Within the courses in this data set, some tunnels had a straight approach, but if a handler chose to wrap the other wing on the jump, the approach became one with some banking on the walls. However, some course designs did not have handling options (like the exit off a dogwalk). When the dogwalk faced the refusal plane of the tunnel, dogs with running contacts had significant issues with the entry (slips, taking of shoulders inside the entry); wet ground conditions compounded the risks for the dogs, including colliding into the closest edge of the tunnel. Dogs with stopped contacts did not face the same issue off the dogwalk exit and the refusal plane approach.

A major issue noted in the data collection was that tunnels often morphed shapes during competition. The phrase “set it and forget it” has significant implications on tunnel incidents, *regardless of type of fixture*. While morphing was most visible on longer tunnels with insufficient tunnel bags, it was also evident with tunnels with plates on the entries and a significant number of tunnel sandbags on the body. With any banking (regardless of location within the tunnel), tunnels appear to morph, some more than others. When the tunnels morphed and developed one or more vertices or changed shape, the tunnel codes were adjusted for the subsequent runs. Vertices are a strong predictor of incidents inside the tunnel, and it is critical that vertices in the tunnels are avoided or corrected. Qualitatively noted by the coders (but not coded quantitatively), there appeared to be a few other factors: incomplete extension of the tunnel at the onset of the course, and insufficient weight in the tunnel bags. Show hosts, judges and competitors should note these prior to course walk-throughs as they create too much give in the tunnel.

More research needs to examine how morphing occurs; however, coders qualitatively noted if insufficient bags are used, then vertices appear to develop near the entry/exit. When plates are used on the ends, the vertices appear to develop in the middle of the tunnel even with substantial bagging of the body. Similarly, over-securing the tunnel may actually prevent ring crews from seeing or being able to pull out any vertices that develop. We recommend that the tunnel mouths are noted on the ground (paint/tape) as best as possible, and ring crews regularly pull and reset the entries to prevent vertices (as discussed earlier, some shifting of tunnel mouths may help dog safety and prevent dogs getting hung-up on or bounced off the exits). Additionally, we recommend organizations not rely on entry-to-entry measures for design requirements. Actual tunnel lengths vary, and the prescribed distance may promote incomplete extension of the tunnels and promote the development of vertices. Rather, the use of the angle of refusal planes may be a better guide with the outside (far) wall of the tunnel fully extended (see Supplemental Materials Figure A).

For trial hosts, the following are some site recommendations. First, grass is the best footing for tunnel incident rates; however, this changes with damp, but not raining, conditions. When the grass becomes wet with active rain or standing water, the risks are not statistically different from dry grass. This might be due to the handlers or the dogs regulating the dog’s speed a bit more or it could be due to a self-selection bias if handlers of dogs who are prone to slips or falls (given their speed or manner of running a course) pull their dog from the run, given the deteriorated conditions. Sand is next best footing. Interestingly, artificial turf performed most poorly for tunnel incidents of the three options, despite it being indoors and dry (in this data set). This may be due to the speeds achieved by the dogs on the artificial turf, thus the force of impact and level of banking within the tunnels; however, future research needs to confirm this.

## Conclusion

This research was conducted to identify risk factors associated with Slips, Falls and Delayed Exits (i.e., slips, falls, significant slow-downs inside the tunnel) to account for base-rates and recall bias and attribution errors associated with a previous tunnel survey based on handler, judge and trainer responses (23). The findings support many assumptions that already exist within the sport, but also directly refute some assumptions and identify some previously unknown risk factors. The results show that tunnel safety is affected by several factors including: the equipment (tunnel interior, wire pitch, length, shape – vertices in particular, types of fixtures), ground type, environmental conditions, course design and handling choices. To that end, the improvement of dog safety while performing the most variable and second most common obstacle in the sport will require efforts by all associated with the sport from equipment manufacturers, trial hosts, judges, trainers and competitors.

## Data Availability

The raw data supporting the conclusions of this article will be made available by the authors, without undue reservation.
