# Supplementary material for: Dog agility tunnel risks for incidents
Source: Front Vet Sci. 2025 Feb 24;12:1547824. doi: 10.3389/fvets.2025.1547824 (PMC11892107; doi:10.3389/fvets.2025.1547824)

Coding Standards

CODES FOR DATA COLLECTION ON TUNNEL RISKS		
Event Level	1	Local Trial
	2	Regionals / Qualifiers for Nationals
	3	Nationals / Qualifiers for Internationals
	4	International
Class Level	1	Levels 0-1 (A0-A1), Grade 1-3, Beginner (UKI), Starters (AAC/USDA)
	1.5	Beginner/Novice mixed class (UKI)
	2	Level 2 (A2), Grades 4-5, Novice (UKI), Advanced (AAC/USDA)
	3	Level 3 (A3), Grades 6-7, Senior/Champ/Open (UKI), Masters (AAC, USDA)
	4	International Qualifiers / International Competition
Tunnel Color Codes	1	Yellow
	2	Light blue (sky blue - see example)
	3	Light purple (see examples)
	4	Light pink
	5	Green
	6	Red
	7	Dark Blue (Royal to Navy blue range - see example)
	8	Dark Purple (see example)
	9	Black
	10	other (specify)
Tunnel Color Patterns	1	solid color throughout
	2	half-and-half (top/bottom different colors)
	3	striped (top, bottom plus strip running lengthwise)
	4	blocked (e.g., rainbow blocks)
Tunnel interior	1	no anti-slip
	2	half-and-half
	3	full anti-slip (note brand)
Tunnel Shape codes - see photos & criteria	1	straight
	2	gentle curve (exit visible from entry)
	3	(-acute (refusal planes intersect at an angle angle)
	4	(-obtuse (refusal planes intersect at a right or obtuse angle)
	5	C curve - ends face same direction but apart as much as possible
	6	U curve - ends face same direction but closer together
	7	J curve - long side with curve at one end
	8	L curve - 90-degree turn anywhere in the tunnel (right angle)
Tunnel Vertex (corner) location	0	No vertex = straight or constant curve
	1	Vertex in entry 1/3 of the tunnel (also use to indicate entry curve for J tunnel)
	2	Vertex in the middle
	3	Vertex in exit 1/3 of the tunnel (also use to indicate exit curve for J tunnel)

Entry/Exit fixtures	1	cinch - screwed into ground
	2	Tunnel bags with peagravel/sand (wide strap)
	3	Tunnel bags with peagravel/sand (narrow strap)
	4	Tunnel bags with water
	5	Plates that grip the ground with tunnel bags over top
Body (not exits) fixtures type	1	cinch - screwed into ground
	2	Tunnel bags with peagravel/sand (wide strap)
	3	Tunnel bags with peagravel/sand (narrow strap)
	4	Tunnel bags with water
	5	Plates that grip the ground with tunnel bags over top
Tunnel bag shape	1	Saddle-bag style (does not hug bottom of tunnel)
	2	Rounded/Cylinder (touches bottom of tunnel not mid)
	3	Triangle style (hugs bottom of tunnel and lower mid)
	4	other (specify)
Ground codes	1	grass
	2	sand
	3	artificial turf (note type - rubber infill / Juta / Other - describe blades of "grass")
	4	other (specify)
Conditions codes	1	dry
	2	damp to wet, but not actively raining
	3	actively raining, wet ground
	4	soaked with standing water
	5	muddy tracks
Approach codes	1	straight approach
	1.5	Handler choice / dog path variance in design results in either straight or angled-open
	2	angled-open (not past refusal plane)
	2.5	Handler choice / dog path variance in design results in either angled-open or closed (blind) approach
	3	closed (blind) approach
Lead on approach	1	n/a - straight tunnel
	2	same lead as tunnel
	2.5	Unknown (handling choice available for either lead on approach)
	3	opposite lead as tunnel
Performance codes	√ 1	Good performance (no slip, fall, or delayed exit)
	o 2	Slip but upright
	● 3	Heard fall/slip/delayed exit, but not seen
	x 4	Fall onto shoulder/hip/side

Supplemental Materials – Tunnel Coding Illustrations and Criteria: Shapes and Approaches

Figure A: Tunnel Shape Codes

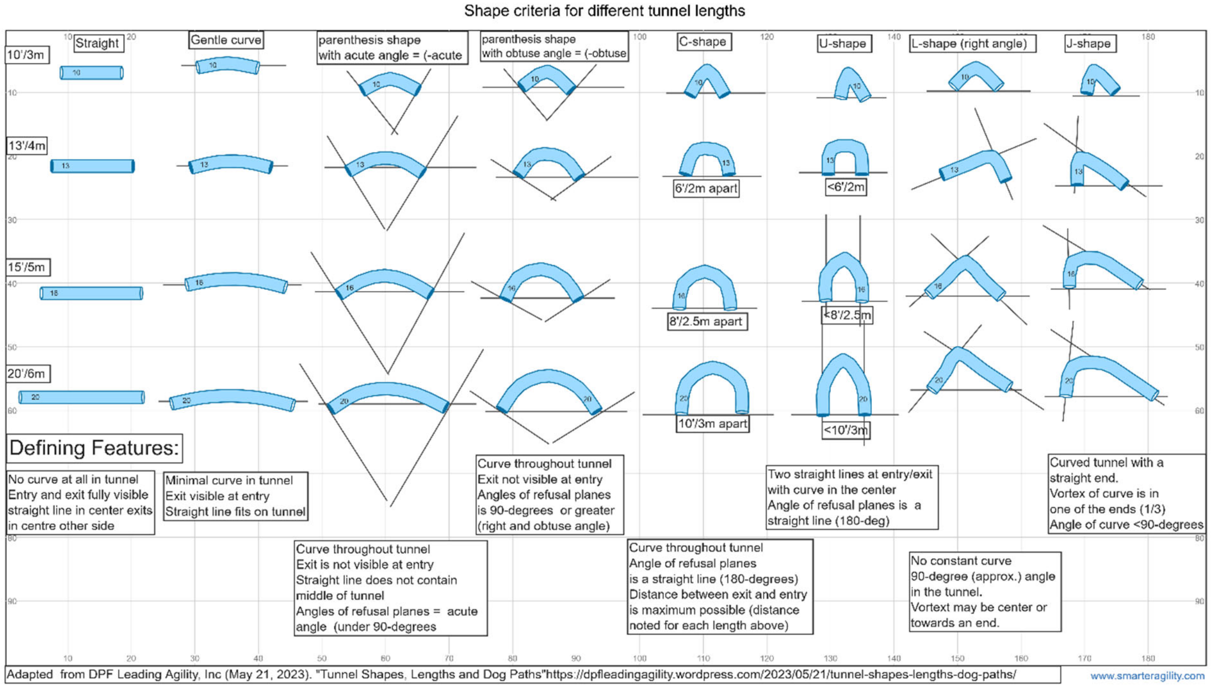

Figure B: Approach Angle Codes

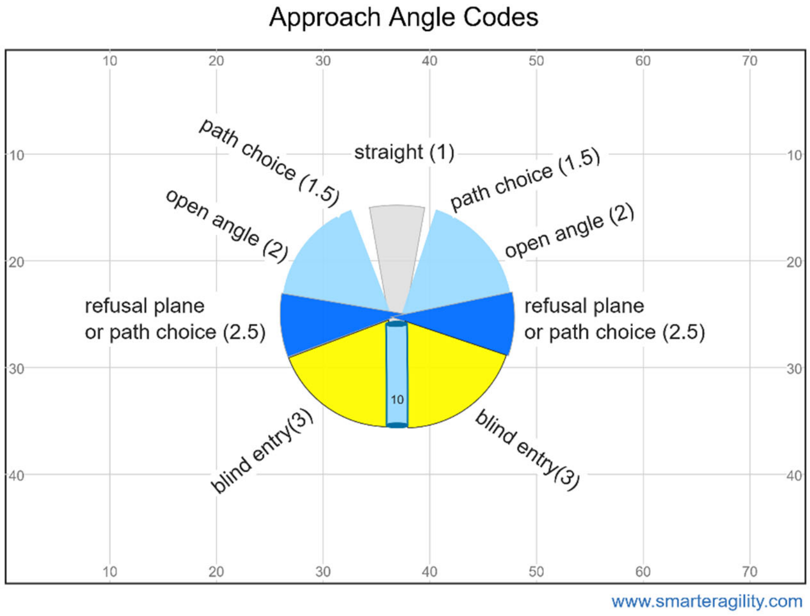

Supplemental Materials – Tunnel Shapes Relative to Dog Path

Figure C: Dog Paths and Tunnel Shapes Illustrations

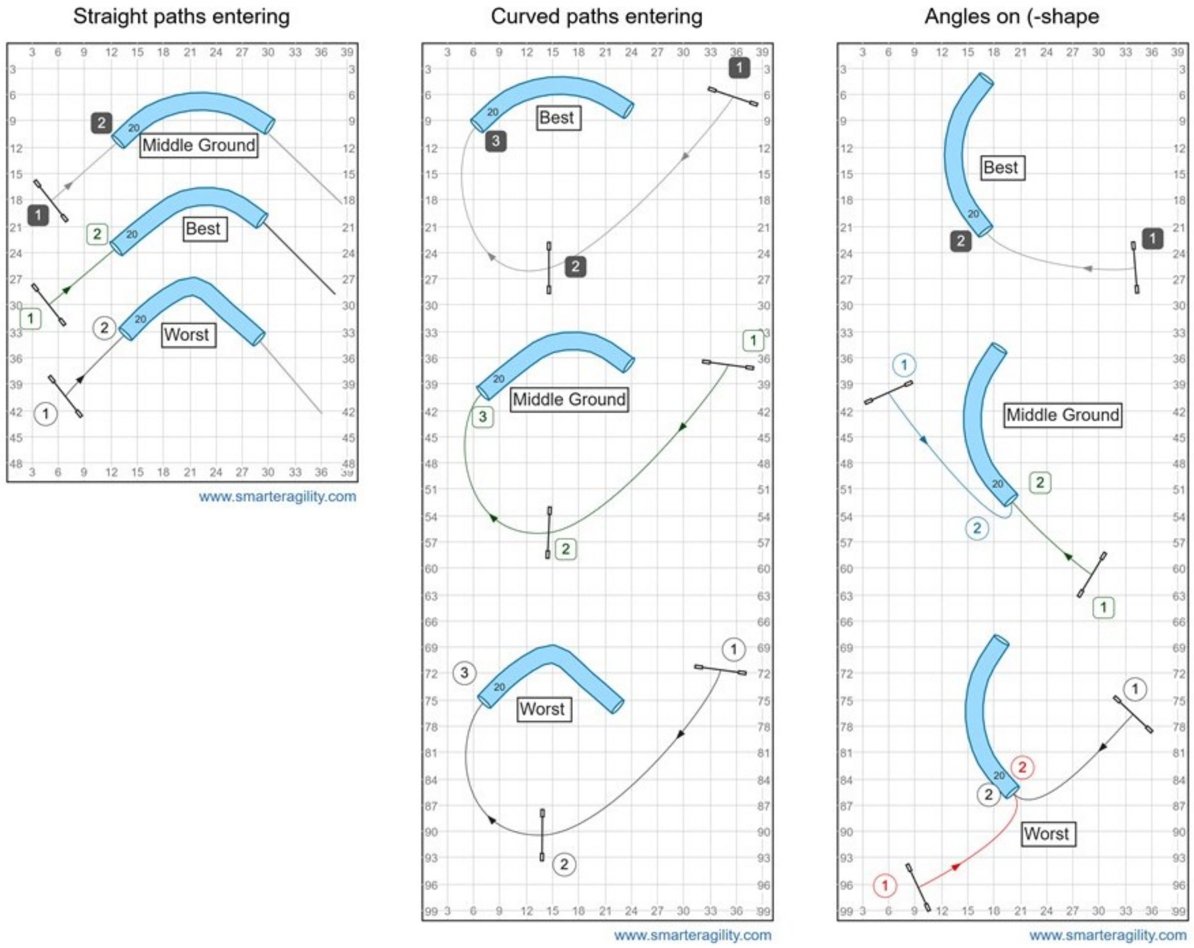

Supplement: Supplementary file 1 [file Data_Sheet_1.PDF]
