# Supplementary material for: Dog agility tunnel risks for incidents
Source: Front Vet Sci. 2025 Feb 24;12:1547824. doi: 10.3389/fvets.2025.1547824 (PMC11892107; doi:10.3389/fvets.2025.1547824)
Supplement: Supplementary file 2 [file Table_1.docx]

**Appendix A.**

Tunnel Sample Descriptive Statistics: N = count of tunnels; there was more than one body fixture type on some tunnels, so sum of % may be greater than 100%.

| **Event Level** | **N (%)** | **Class Level** | **N (%)** |
| --- | --- | --- | --- |
| Local | 285 (50.7) | Levels 0-1, Grade 1-3, Beginner (UKI), Starters (AAC/USDA) | 40 (7.1) |
| Regional | 35 (6.2) | Beginner/Novice mixed class (UKI) | 52 (9.2) |
| National | 125 (22.2) | Level 2 (A2), Grades 4-5, Novice (UKI), Advanced | 38 (6.8) |
| World’s | 118 (20.9) | Level 3 (A3), Grades 6-7, Senior/Champ/Open (UKI), Masters | 232 (41.1) |
| **Conditions** | **N (%)** | World Qualifiers/Worlds | 202 (35.8) |
| Dry | 369 (65.4) | **Ground (Footing)** | **N (%)** |
| Damp-to-Wet, No Rain | 123 (21.8) | Grass | 307 (54.4) |
| Active Rain Wet | 56 (9.9) | Sand | 156 (27.7) |
| Soaked with Standing Water | 12 (2.1) | Artificial Turf | 101 (17.9) |
| Muddy Tracks | 4 (0.7) | **Vertex** | **N (%)** |
| **Tunnel Interior** | **N (%)** | None | 529 (93.8) |
| No Anti-Slip | 87 (15.4) | Entry | 12 (2.2) |
| Half Anti-Slip | 10 (1.8) | Middle | 15 (2.7) |
| Full Anti-Slip | 564 (82.8) | Exit | 10 (1.8) |
| **Tunnel Colour** | **N (%)** | **Tunnel Shape** | **N (%)** |
| Yellow | 158 (28.2) | Straight | 154 (27.3) |
| Light Blue | 33 (5.9) | Gentle Curve-Exit Visible | 126 (22.3) |
| Light Purple | 68 (12.1) | (-curve: Refusal planes acute angle | 163 (28.9) |
| Light Pink | 54 (9.6) | (-curve: Refusal planes obtuse angle | 83 (14.7) |
| Green with Yellow stripe | 7 (1.3) | C-Shape | 8 (1.4) |
| Red | 156 (27.9) | U-Shape | 3 (0.5) |
| Dark Blue | 54 (9.6) | L-Shape | 18 (3.2) |
| Dark Purple | 21 (3.8) | J-Shape | 6 (1.1) |
| Yellow Top, Black Bottom | 9 (1.6) | S-Shape | 3 (0.5) |
| **Colour Pattern** | **N (%)** | **Tunnel Length** | **N (%)** |
| Solid | 544 (97.1) | 10’ (3m) | 52 (9.2) |
| Half-and-Half | 9 (1.6) | 13’ (4m) | 8 (1.4) |
| Stripe splitting top and bottom | 7 (1.3) | 15’ | 136 (24.1) |
| **Entry/Exit Fixture Type** | **N (%)** | 16’ (5m) | 128 (22.7) |
| Cinch – Screwed into ground | 66 (11.7) | 19’ | 8 (1.4) |
| Bags: Peagravel/Sand, Wide Straps | 397 (70.4) | 20’ (6m) | 232 (41.1) |
| Bags: Peagravel/Sand,NarrowStraps | 6 (1.1) | **Wire Pitch** | **N (%)** |
| Bags: Water filled | 0 (0) | 4” | 238 (43.5) |
| Hugger/Plate plus Bag with Peagravel/Sand, Wide Straps | 95 (16.8) | 6” | 296 (54.1) |
|  |  | 7” | 2 (0.4) |
| **Body Fixture Type** | **N (%)** | 8” | 11 (2.0) |
| Cinch – Screwed into ground | 54 (9.6) | **Approach** | **N (%)** |
| Bags: Peagravel/Sand, Wide Straps | 509 (90.4) | Straight | 223 (39.5) |
| Bags: Peagravel/Sand,NarrowStraps | 62 (11.0) | Straight-Open (Handler choice: 1-2) | 18 (3.2) |
| Bags: Water filled | 3 (0.5) | Open-Angled Approach | 275 (48.8) |
| Hugger/Plate plus Bag with Peagravel/Sand, Wide Straps | 0 (0) | Refusal Plane (Handler-choice: 2-3) | 14 (2.5) |
|  |  | Blind-Closed Approach | 34 (6.0) |
|  |  | **Expected Lead on Approach** | **N (%)** |
|  |  | Not Relevant (Straight tunnel only) | 150 (26.6) |
|  |  | Same Lead as Tunnel Curve | 293 (52.0) |
|  |  | Opposite Lead as Tunnel Curve | 90 (16.0) |
|  |  | Unknown Lead (Handler Choice) | 31 (5.5) |
